# Lake- and groundwater-associated alteration of the olivine-rich Margin unit in Jezero crater, Mars

**DOI:** 10.1038/s43247-026-03997-9

**Published:** 2026-09-21

**Authors:** Candice C. Bedford, Eleni Ravanis, Roger C. Wiens, Elise Clavé, Julene Aramendia, Juan Manuel Madariaga, Eleanor Moreland, Stephanie Connell, Alexander Jones, Briony Horgan, Olivier Forni, Bradley Garczynski, Susanne Schröder, Kenneth Williford, Linda Kah, Kathryn Stack, Lucia Mandon, Agnés Cousin, Pierre Beck, Erwin Dehouck, Clément Royer, Adrian Brown

**Affiliations:** 1 Earth, Atmospherics and Planetary Science, Purdue University, West Lafayette, IN USA; 2 Hawaiʻi Institute of Geophysics and Planetology, University of Hawai’i at Mānoa, Honolulu, HI USA; 3 Institute of Space Research, DLR, Berlin, Germany; 4 Department of Analytical Chemistry, University of the Basque Country (UPV/EHU), 48940 Leioa, Spain; 5 Rice University Department of Earth, Environmental and Planetary Sciences, Houston, TX USA; 6 Department of Earth Science and Engineering, Imperial College London, London, UK; 7 Institut de Recherche en Astrophysique et Planétologie, Université de Toulouse, CNRS, CNES, Toulouse, France; 8 Western Washington University, Bellingham, WA USA; 9 Blue Marble Space Institute of Science, Seattle, WA USA; 10 Earth, Environmental, and Planetary Sciences, University of Tennessee, Knoxville, TN USA; 11 Jet Propulsion Laboratory, Pasadena, CA USA; 12 Université Grenoble Alpes, CNRS, IPAG, Grenoble, France; 13 Université Lyon 1, ENS de Lyon, Université Jean Monnet, CNRS, LGL TPE, UMR 5276, Villeurbanne, France; 14 Institut d’Astrophysique Spatiale, Université Paris-Saclay, Orsay, France; 15 Plancius Research, Severna Park, MD USA

**Keywords:** Geochemistry, Geochemistry

## Abstract

The Margin unit in Jezero crater, Mars, was explored by NASA’s *Perseverance* rover as orbital data suggested it may represent an ancient lake shoreline. Using SuperCam data, we evaluate the origin and alteration history of this enigmatic unit. Here, we show that the Margin unit explored at high elevations (>−2350 m) has a texture and chemistry indicative of a slow-cooled, crystalline, olivine-rich rock without significant water exposure. In contrast, exposures situated below the hypothesized Jezero paleolake’s second terrace level reveal a complex history of water-rock interaction, with signs of physical reworking near Neretva Vallis and the Western fan. We identify three main alteration episodes: first, neutral-to-alkaline CO_2_-rich fluids circulated within the bedrock, forming carbonate-rich ridges upon erosion. Then, exposure to the Jezero paleolake waters or changes in groundwater fluids remobilized carbonate and precipitated silica in the secondary pore spaces. Finally, late-stage hydrothermal fluids flowed through younger fractures, precipitating fluorite-bearing, Ca-sulfate mineral veins. This sequence of multiple fluid events driven by groundwater activity and exposure to the lake reinforces the Margin unit as a locality of astrobiological interest.

## Abstract (web summary)

Textural, mineralogical, and chemical analyses of the Jezero crater Margin unit from Perseverance’s SuperCam identify multiple episodes of aqueous alteration driven by early carbon-rich fluids, paleolake/groundwater interaction, and late-stage hydrothermal activity.

## Introduction

**Fig. 1:**
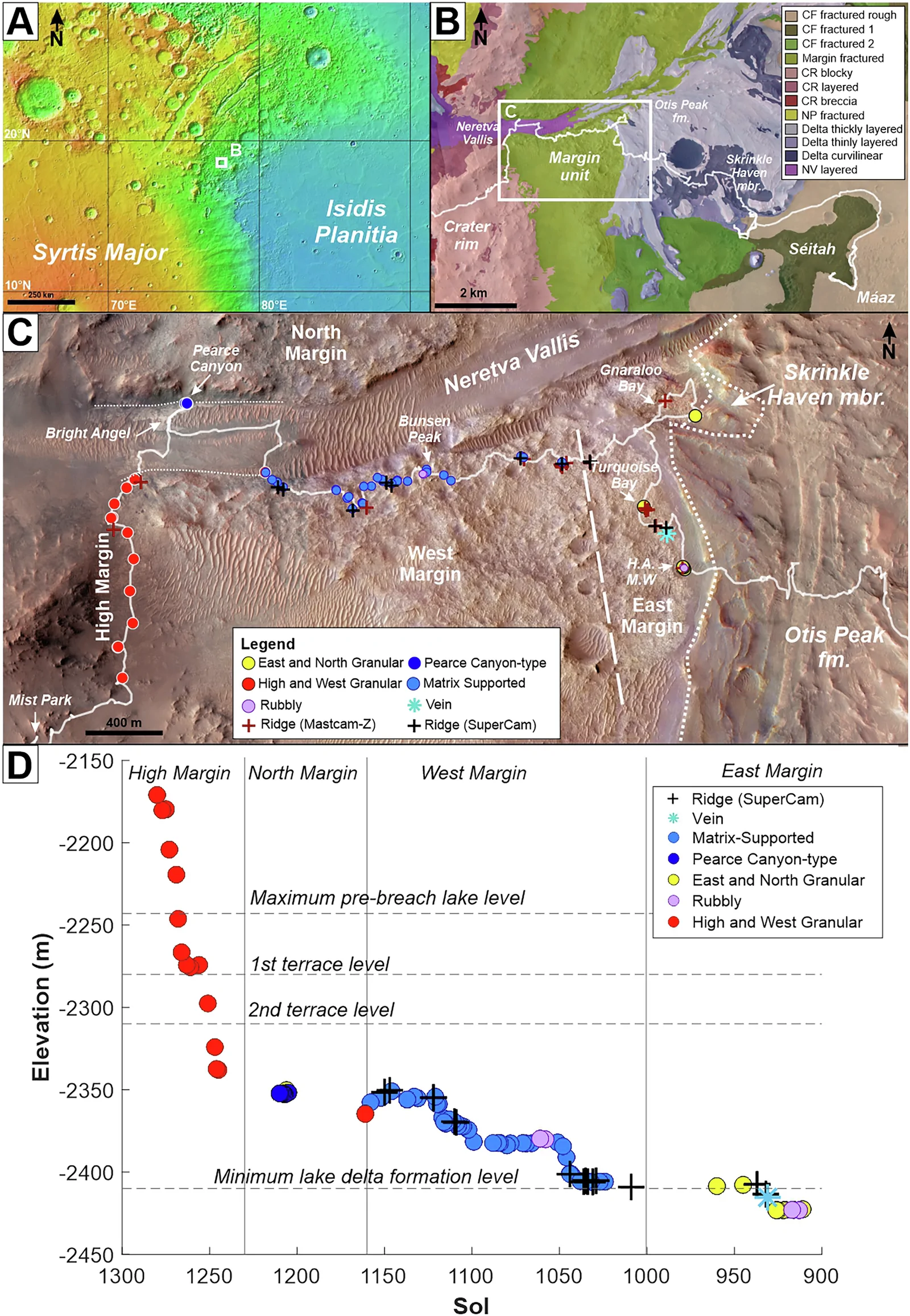
Regional and local context of the Margin unit and its associated bedrock textures and alteration features as defined in this manuscript and identified along the *Perseverance* rover traverse. **A** A regional topographic map generated by the Mars Orbiter Laser Altimeter (MOLA) instrument onboard the Mars Global Surveyor. Red relates to areas of high elevation (up to 2.1 km); blue relates to areas of low elevation (up to −4.0 km). Latitude and longitude are provided on the image, and the white square marked ‘B’ shows the location of Jezero Crater and subpanel B. This image was generated using JMARS^70^. **B** The geologic map of the Jezero crater Western Fan by Stack et al. 2020^71^ is colored according to orbitally identified units (CF Crater Floor, CR Crater Rim, NP Nilli Planum, NV Neretva Vallis). Units of interest to this study and features of interest are annotated, such as Neretva Vallis, the main inlet channel of the Western fan. The white square marked ‘C’ shows the location of subpanel C. The rover traverse is shown as a solid white line. This image and that in subpanel C were produced using the 007 operational HiRISE mosaic^72,73^ with the CAMP (Campaign Analysis Mapping and Planning) GIS tool^58,60^. **C** Shows the rover traverse across the Margin unit (white line), approximate geological boundaries between the units of the Western fan and those of the Margin Unit (bold dotted white line), and the approximate geological boundaries between the Margin unit and the Bright Angel formation (dotted white line). The divisions of the East and West Margin are marked as a dashed white line and key locations are shown. This map also shows the spatial distribution of SuperCam target textural groups identified in this study except for targets whose textures are obscured by dust/regolith cover or poor focus/lighting conditions and marked as “unknown” (see Methods). As SuperCam ridge observations are restricted according to rover resources, locations of ridge features are shown here as black crosses if targeted by SuperCam and brown crosses if seen in Mastcam-Z images but not targeted by SuperCam. This is to provide clearer context to the spatial extent of these features. **D** provides the SuperCam target textural classes in context with elevation. Sol (Martian mission day) number is displayed on the x-axis as a proxy for distance and oriented to match the traverse direction of the map. Meanwhile, elevation refers to elevation from the Martian areoid and lake levels^33^ are noted. HAMW Hans Amundsen Memorial Workspace.

The NASA Mars 2020 *Perseverance* rover landed in Jezero Crater in February 2021 to investigate the geological record and look for signs of ancient life^1^. Jezero crater is 45 km in diameter and is situated on the northwestern side of the 1200 km Isidis impact basin, and northeast of the Syrtis Major volcanic province near a region known as Nilli Fossae (Fig. 1). The Nilli-Syrtis region has one of the most widespread occurrences of olivine and carbonate on Mars^2–5^. Previous research hypothesized the olivine-carbonate-rich rocks of Jezero crater to be related to the regional olivine-rich unit^6^. A narrow area with particularly strong carbonate signatures was identified within the inner margin of Jezero crater, close to the western inlet valley^7^, now informally named the “Margin unit”. The Mars 2020 mission considered the Margin unit as a location of high importance to explore and sample with the Perseverance rover due to its potential for the carbonates to have been precipitated in a lake shore environment^1,7^, an environment favorable to the preservation of biosignatures.

During the Mars 2020 mission, the Margin unit was divided into four localities (Fig. 1): East Margin (investigated on mission days or “sols” 911–1000), West Margin (sols 1021–1161), North Margin (sols 1192–1211), and High Margin (sols 1245–1280). The East, West, and North Margin correlate to the boundaries of the Margin unit previously mapped from orbit^7^. The High Margin is an informal extension of these previously mapped boundaries that corresponds to the apparent continuation of the olivine-bearing rocks of the Margin unit to elevations above −2300 m^8^. *Perseverance* data also revealed olivine-bearing rocks at Mist Park, in the inner crater rim (Fig. 1C). This paper excludes that region, which is beyond the continuous Margin unit deposit and within an area of lithological diversity potentially relating to impactites.

The East Margin unit largely consists of low-lying bedrock slabs with cm-scale layering and possible cross-beds^8–10^ and is the lowest part of the Margin unit investigated, at elevations of −2423 and −2411 m at Hans Amundsen Memorial Workspace (HAMW), Turquoise Bay, and Gnaraloo Bay (Fig. 1C). Two abrasion patches with proximity science observations were acquired at HAMW and Turquoise Bay named Amherst Point and Bills Bay, respectively. Two samples were also acquired from these locations (Pelican Point at HAMW and Lefroy Bay at Turquoise Bay) for a potential sample return mission to Earth.

The West Margin (elevations between −2410 and −2352 m) has thicker (decimeter to meter) layered, massive beds with a pitted or wind-abraded and polished surface compared to the East Margin^8^. After the Bunsen Peak workspace (sol 1069), where the Castle Geyser patch was abraded and a Comet Geyser sample was collected, the outcrop appeared extensively fractured and ventifacted. Towards the end of the West Margin unit traverse, on the descent to Neretva Vallis, another abrasion patch was acquired, called Old Faithful Geyser.

The North Margin unit was investigated at the northern side of Neretva Vallis in the Bright Angel area to determine the relationship between the Margin unit and the largely fine-grained, extensively altered material of the Bright Angel fm^11,12^. The stratigraphically lowest exposure of the Margin unit contains a matrix-supported, heterolithic, clast-rich layer that was investigated in detail at Pearce Canyon. Above this conglomerate, the outcrop has a similar granular appearance to the Margin unit investigated south of Neretva Vallis. No abrasion patches were acquired here, and only remote sensing compositional data were gathered from this area between elevations −2350 to −2352 m.

The High Margin unit covers the largest range in elevation (−2338 to −2159 m) and contains fractured, ventifacted bedrock that appears granular^8,10^. The Emerita Mesa abrasion patch was acquired here, and no samples were taken.

*Perseverance*’s remote geochemistry instrument, SuperCam, acquires quantitative chemical data of targets between 2 and 6.5 m of the rover mast along with high-resolution images and reflectance spectra^13,14^. These remote sensing capabilities have enabled SuperCam to analyze chemistry across >185 bedrock targets across the Margin unit, including alteration features. In this manuscript, we show evidence from several localities that the Margin unit was emplaced as a crystalline, igneous rock with signs of sedimentary reworking. We also show that the Margin unit’s alteration was likely enhanced through exposure to the Jezero paleolake and groundwater fluids.

## Results

### Cluster results

**Fig. 2:**
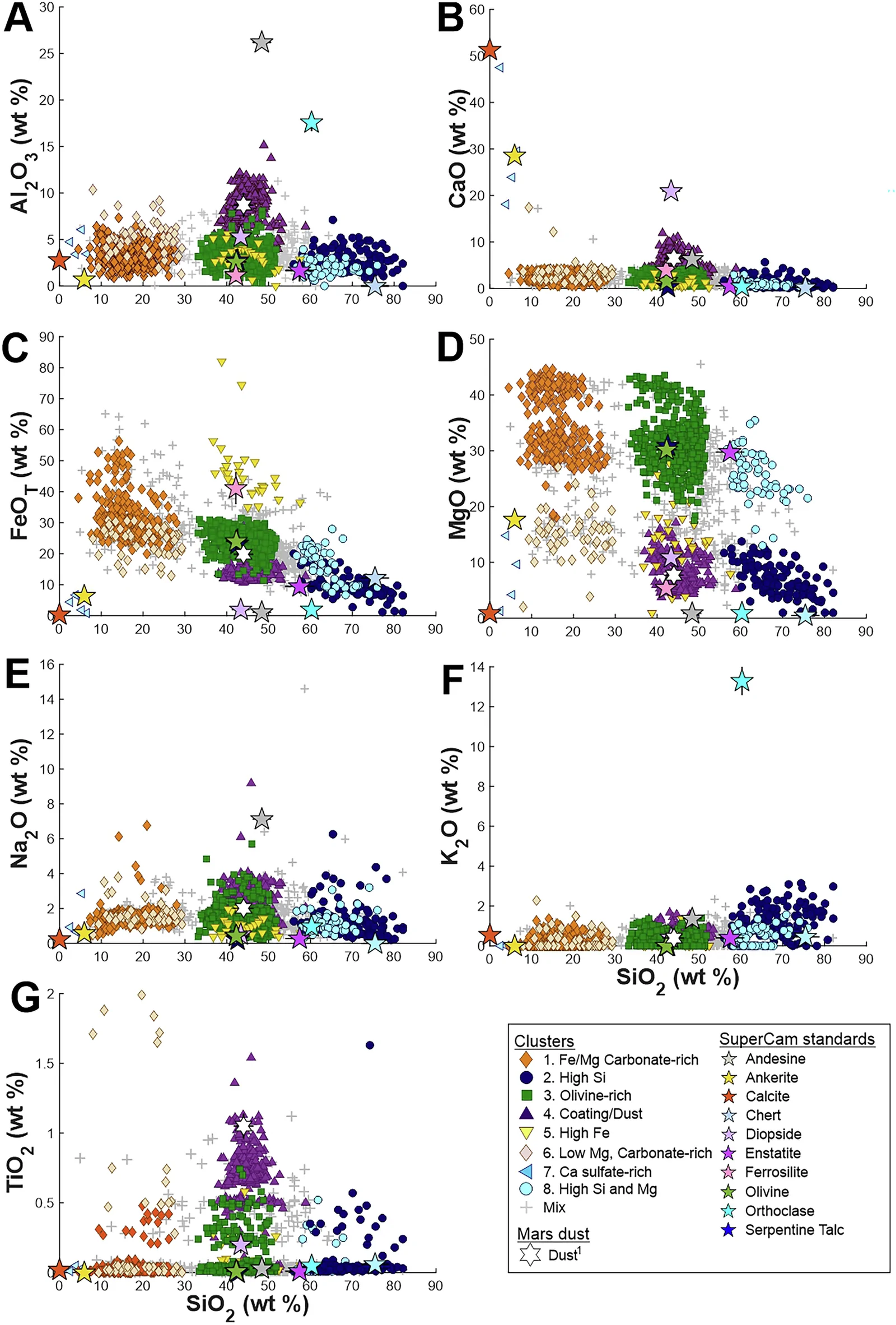
Plots of SuperCam LIBS cluster results. These element biplots show the spread in composition of **A** Al_2_O_3_, **B** CaO, **C** FeO_T_, **D** MgO, **E** Na_2_O, **F** K_2_O, and **G** TiO_2_ against SiO_2_ on the x-axis for all LIBS data used in this study. Data are plotted alongside select MOC data of SuperCam onboard standards taken on sol 1200 (see supplementary information for list of standards and their composition) and the composition of Mars dust determined by 1) Lasue et al.^74^. Data relating to the “Mix” cluster are data that had low silhouette scores indicative of a poor fit to all clusters. 2σ is shown as black lines for SuperCam standards, though many are smaller than the symbol itself.

The SuperCam Margin unit bedrock dataset was divided into eight clusters through a hierarchical agglomerative cluster analysis (Fig. 2 and S1). Overall, 356 data points (22% of the dataset) had a weak relationship to their assigned cluster (see Methods) and were placed into their own “mixed” group (Fig. 2). Clusters were best defined according to differences in SiO_2_, MgO, and FeO_T_ (Fig. 2C and D), but some notable variation exists in Al_2_O_3_, CaO, and TiO_2_ (Fig. 2A, B, G). The following is a breakdown of the clusters:

1. Cluster 1 (17% of the data) is characterized by low SiO_2_ (16.3 ± 4.5 wt% mean ± stdev) and TiO_2_ (0.04 ± 0.08 wt%) values, but high MgO (35.1 ± 6.0 wt%) and C scores (Fig. S2), with moderately high FeO_T_ (31.6 ± 6.8 wt%). The low SiO_2_ abundances, high MgO and FeO_T_ abundances, and elevated C scores suggest that this cluster is related to a Mg and Fe carbonate-rich component, like that identified in VISIR (Fig. S9), Raman^15^, and proximity science results^8^. As such, this cluster is interpreted as a Fe/Mg-rich carbonate cluster.
2. Cluster 2 (7% of the data) has high SiO_2_ with an average of 69.3 ± 6.1 wt%. MOC values for K_2_O are also high, but this is not notably reflected in the ICA scores (Fig. S7) and is considered an error with the MOC at high SiO_2_ abundances. All data points of this cluster relate to bedrock observations consistent with a silica-rich phase detected by proximity science instruments in the bedrock^8^.
3. Cluster 3 (25% of the dataset) has moderate SiO_2_ (44.1 ± 4.6 wt%) and FeO_T_ (24.1 ± 3.4 wt%), but high MgO (31.6 ± 5.3 wt%). The composition of this cluster is scattered around the olivine SuperCam standard. Olivine has been identified as a major contributor to the Margin unit bedrock investigated by proximity science instruments at abrasion patches^8^ and other SuperCam techniques^15^, which corroborate the cluster analysis results.
4. Cluster 4 (20% of the data) contains the highest abundances of Al_2_O_3_ (8.5 ± 2.0 wt%), TiO_2_ (0.7 ± 0.2 wt%), and Na_2_O (2.6 ± 0.8 wt%), elevated CaO (5.3 ± 1.7 wt%), with low MgO abundances (7.4 ± 2.7 wt%) and moderate SiO_2_. This cluster correlates to the presence of coatings that vary in thickness and have a composition close to Martian dust (Fig. 2). This cluster likely corresponds to a thick dust cover or a coating. As coatings are a late-stage alteration feature that covers a wide variety of rock types in Jezero crater^16^, these data are removed from our analysis of the Margin unit.
5. Cluster 5 (2% of the data) has moderate SiO_2_ abundances (44.5 ± 5.1 wt%) with the highest FeO_T_ abundances of any cluster (46.4 ± 10.3 wt%) and a high total sum of oxides ( > 95 wt%), suggesting an iron-rich phase. As such, this cluster is referred to as the Fe-rich cluster.
6. Cluster 6 (3% of the data) has a similar composition to Cluster 1 but exhibits scatter towards higher TiO_2_ abundances and much lower MgO (15.0 ± 4.3 wt%) and low totals (70.0 wt% average). Two populations of carbonate were identified in the Margin unit in Raman and LIBS data^15^, one of which has a low Mg# (0.2 to 0.4) similar to that of this cluster (0.4 ± 0.9). As such, we refer to this cluster as low-Mg, carbonate-rich.
7. Cluster 7 (4 data points) also has low SiO_2_ abundances (4.6 ± 1.7 wt%), but this time contains high abundances of CaO (29.8 ± 12.7 wt%) and a low total sum of oxides (51.0 ± 7.5 wt%), indicating the high abundance of elements not quantified in the MOC data. VISIR data suggests the presence of sulfate (Fig. S11), and molecular CaF peaks were detected in these LIBS data (Fig. S10). This cluster is interpreted to represent a significant Ca-sulfate contribution, with fluorite.
8. Cluster 8 (3% of the dataset) has a similarly high abundance of SiO_2_ (63.7 ± 4.4 wt%) as Cluster 2 (Fig. 2); however, it also has high MgO (25.5 ± 4.4 wt%) and higher FeO_T_ (16.0 ± 5.6 wt%). This indicates a higher contribution of MgO- and FeO_T_-bearing minerals to the silica-rich part of the bedrock. Proximity science results confirm the presence of low Ca-pyroxenes in the Margin unit^8^, although these are fewer in abundance than the olivine grains, which may support the few detections in the cluster analysis. Alternatively, this cluster could relate to a fine-scale mixture of silica with olivine grains, given its trend to the high silica Cluster 2. As such, we refer to this cluster as High Mg, Si-rich.

### Bedrock textures

Bedrock textures in the Margin unit were classified according to locality, grain size, grain relationships, rock fabric (e.g., the presence or absence of layering/laminations), and the presence of fine-grained or amorphous interstitial material (matrix). These terms are used to remain relatively agnostic of formation conditions of the rock.

**Fig. 3:**
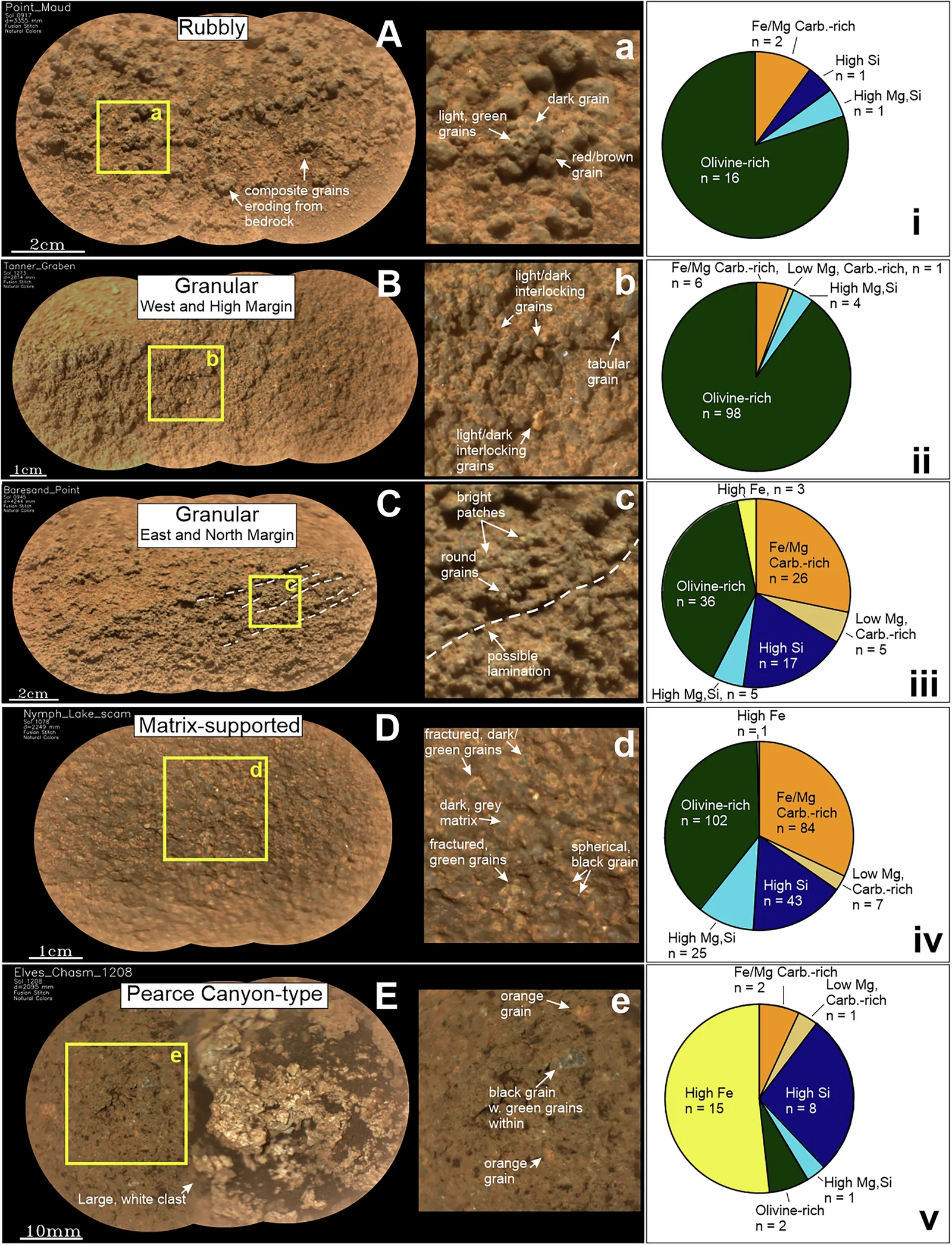
Bedrock textures with SuperCam cluster results showing visual and mineralogical differences between textural classes. All inset images (**A–E**) are set to the same scale (2 cm across). Pie charts on the right indicate the frequency of occurrence of each compositional cluster within the given textural class; the numbers in the pie charts represent the number of data points each wedge represents. Figure A) shows an example of a Rubbly class target (Point_Maud, sol 917) with inset **A** highlighting the features within the composite grains eroding from the bedrock. Figure B) provides an example of the relatively angular, granular texture observed in the West and High Margin unit (target Tanner_Graben, sol 1273) with inset **B** zooming in to highlight interlocking grain relationships and subhedral shapes. Figure C) shows an example of the granular texture seen in both the East and North Margin unit with inset **C** zooming into the possible laminations visible in the Baresand_Point (sol 945) target. **D** provides an example of the matrix-supported texture of the West Margin and **E** shows the Pearce Canyon-type texture with a variety of clast colors, sizes, and shapes. Carb. is shorthand for carbonate.

**Fig. 4:**
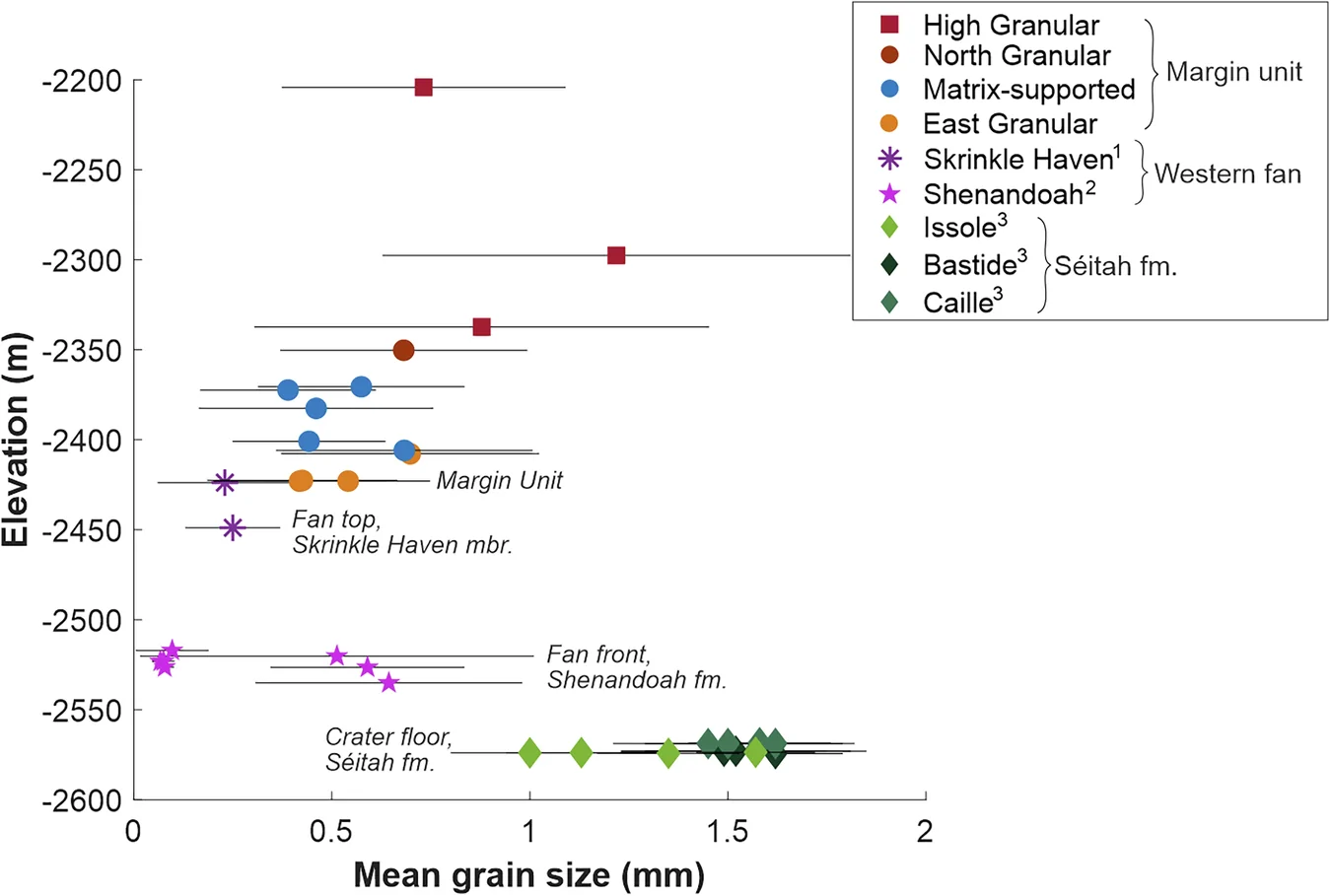
Grain size distributions of different textural classes across the Margin unit with elevation. Points show median grain size values with whiskers indicating 1σ. For the Margin unit data acquired here, the Matrix-supported dataset consists of *n* = 941 measured grains with East Granular *n* = 843, North Granular *n* = 200, and High Granular *n* = 498 (see Table S1 in supplementary information for a breakdown of grain size statistics for each target). Reference grain sizes from previous units are provided for comparison. 1) Ives et al., 2025 ^75^., 2) Stack et al., 2024^76^, 3) Wiens et al., 2022^51^.

The Granular textural class is grain-supported, containing grains of approximately equal size between 0.05 – 10 mm, but the size and shape of the grains vary across the four Margin regions. In all regions, tan to light-toned, interstitial material is common around grain edges, while the grains themselves range in color from very dark green to translucent pale green, reddish-brown, and white (Fig. 3B, C). The West and High Margin Granular targets have grains with a greater apparent angularity to them than the grains comprising the East and North Margin Granular rocks (Fig. 3 and S14). Grain sizes across each raster for the High Margin Granular class are 0.73 ± 0.36 mm to 1.22 ± 0.59 mm (Fig. 4). Granular targets in the East and North Margin are finer-grained. East Margin Granular grain size distributions vary with a mean grain size of 0.42 ± 0.22 mm to 0.70 ± 0.33 mm (Fig. 4). Several East Margin Granular class targets exhibit centimeter-scale layering at the outcrop scale^10^. The North Margin Granular bedrock is texturally very similar to East Margin Granular targets with a similar grain size of 0.68 ± 0.31 mm and decimeter-scale, planar layering (Fig. S13).

The Matrix-supported textural class contains grains that are resolvable in RMI images entrained within a matrix that appears fine-grained below the finest resolvable grain size (0.06 mm/pixel for most targets). Only grain sizes coarser than the resolution of three pixels (0.18 mm) are measured^17^ and share a similar grain size to the East Margin Granular grain size with a mean of 0.39 ± 0.22 mm to 0.68 ± 0.32 mm (Fig. 4). Measured grains are commonly dark green to pale green, frequently surrounded by a pale, yellow rim and can be rounded or angular where fracturing has occurred (Fig. 3D). Matrix is present as dark, gray patches, surrounding the coarser grains (Fig. 3D). This class primarily occurs in the West Margin.

The Rubbly bedrock class occurs as occasional, recessive layers in the East and West Margin containing coarse, subangular, composite grains actively eroding into the surrounding regolith. Measured grain sizes have means of 0.87 ± 0.94 mm to 1.02 ± 1.06 mm.

Finally, the Pearce Canyon-type class is matrix-supported and contains a relatively large grain size range, encompassing grains in the matrix that are too fine to resolve with the RMIs ( < 0.07 mm) and clasts that are larger than the RMI field of view (Fig. 3). The matrix appears tan-brown with abundant pits or grooves. Clasts are polymict and range from smooth, subrounded, light orange clasts; bright white, angular to curved clasts; dull white, nodular, rounded clasts; and dark, irregular patches with pits and green minerals entrained within. Many clasts exhibit orange rims. This textural class is an isolated outcrop ~0.5 m topographically above the unconformity with the Bright Angel Formation, in the Northern Margin.

### Alteration features

**Fig. 5:**
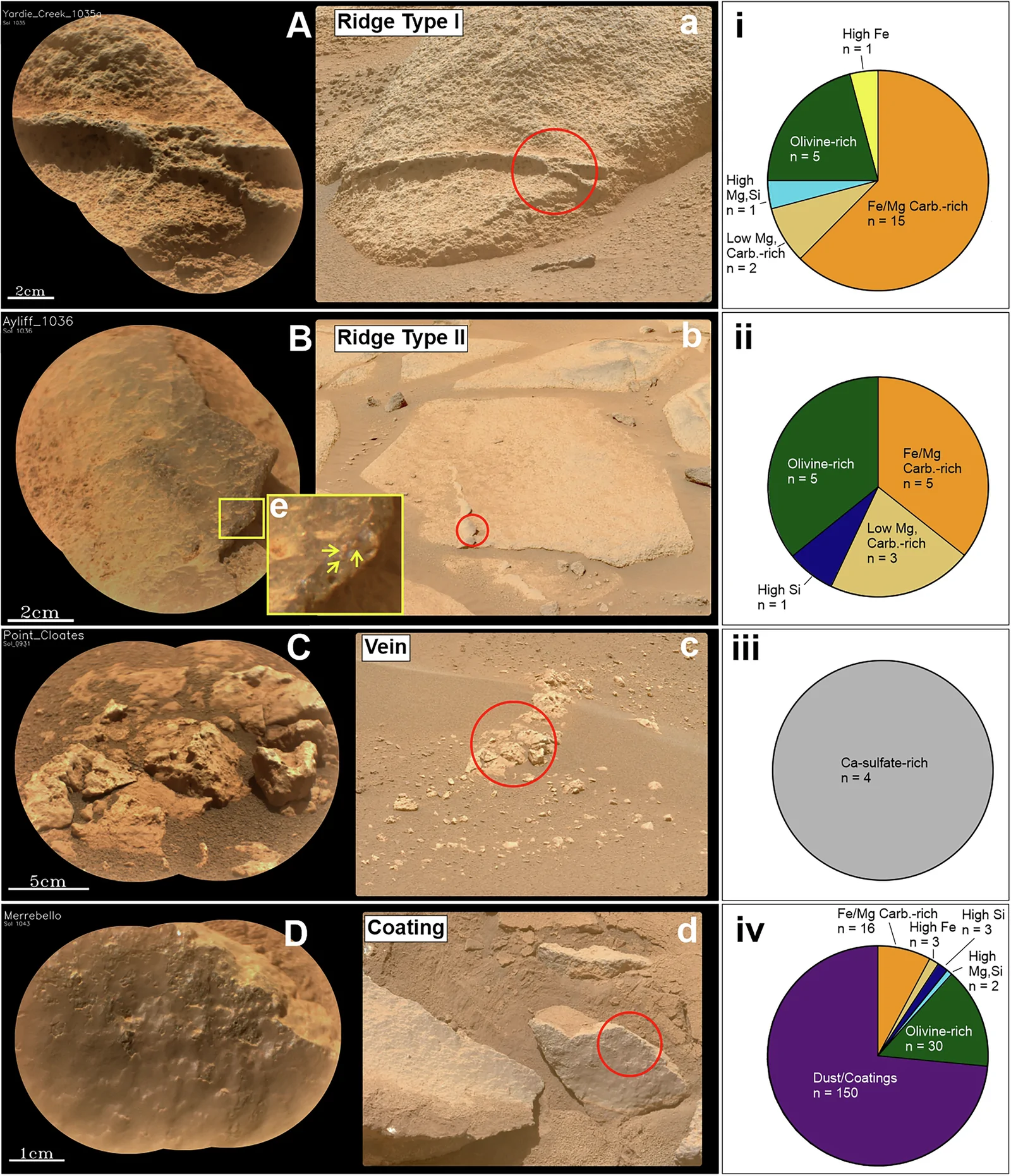
Alteration feature textures and their associated SuperCam cluster results. SuperCam RMIs (**A–D**) are shown alongside larger context Mastcam-Z images of these alteration feature targets, and their associated cluster pie charts (i-iv) indicating the relative abundances of the compositional types (Fig. 2). Mastcam-Z images used include **A** ZCAM09048 sol 1546, **B** ZCAM09038 sol 1032, **C** ZCAM08939 sol 931, and **D** ZCAM09062 sol 1043. Numbers on the pie charts represent the number of data points within each cluster. Yellow arrows in **e** point to green/yellow, round grains within the gray matrix. Carb. is shorthand for carbonate.

**Fig. 6:**
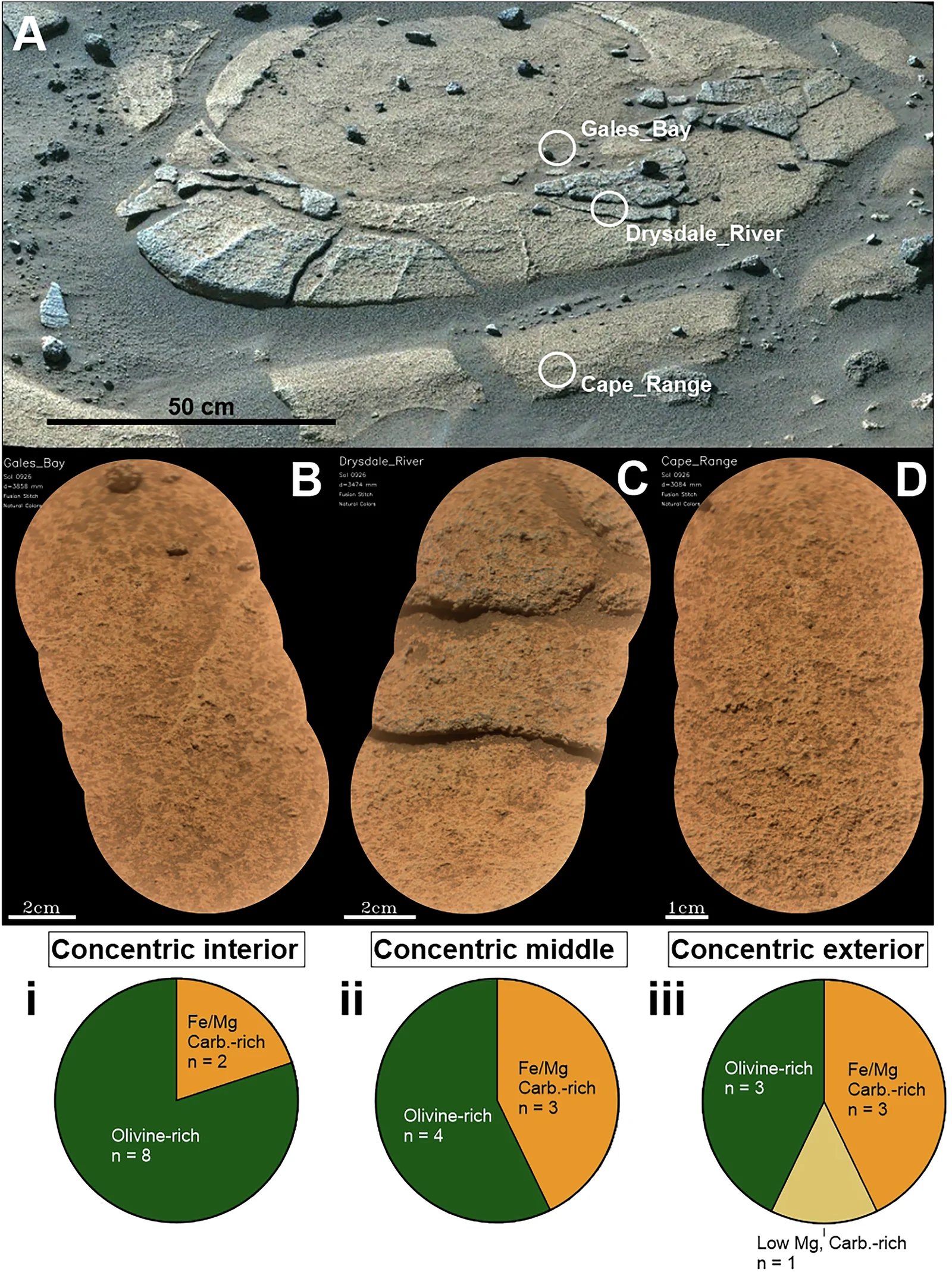
SuperCam cluster results and images of the concentric feature investigated at HAMW. **A** Mastcam-Z image mosaic ZCAM08932 (sol 925) annotated to show SuperCam target locations. Mastcam-Z images are enhanced using a linear stretch. SuperCam fused RMI mosaics of Gales Bay (**B**), targeting the interior of this feature, Drysdale River (**C**), targeting the middle of this feature, and of Cape Range (**D**), targeting the exteriormost part of this feature. Pie charts i) to iii) show the corresponding cluster results of each target shown in (**B–D**). Carb. is shorthand for carbonate.

Four morphologies of aqueous alteration features have been identified in the East and West Margin bedrock (Figs. 5, 6) with coatings present across all Margin localities. Ridges are the most common alteration feature seen in the East and West Margin (Fig. 1C, D). These ridges extend across fractures in the bedrock (e.g., Fig. 5B), have multiple orientations inconsistent with layering, which in some cases cross or branch from one another (e.g., Fig. 5A) and are present in two subtypes. Ridge type I has a similar surface roughness to the surrounding bedrock and appears to slightly protrude from the bedrock. Meanwhile, Ridge type II, strongly protrudes from the bedrock with a fin-like edge, and occasionally small, green grains can be seen in the otherwise fine-grained feature (Fig. 5e).

Large, concentric features have been identified in the East Margin, with one investigated by the SuperCam instrument (Fig. 6). These features are associated with the fractures in the bedrock and can themselves have ridges oriented perpendicular to the feature edge (Fig. 6A).

SuperCam in the East Margin unit analyzed a light-toned mineral vein (Point Cloates, Fig. 5C). This linear feature is disaggregated and has an uncertain relationship to the surrounding bedrock due to regolith cover (Fig. 5C). However, Navcam images show that it extends several meters to the northeast and east, suggesting it is in place and potentially part of a larger vein network in this area (Fig. S17).

### Constraints on the origin of the Margin unit

**Fig. 7:**
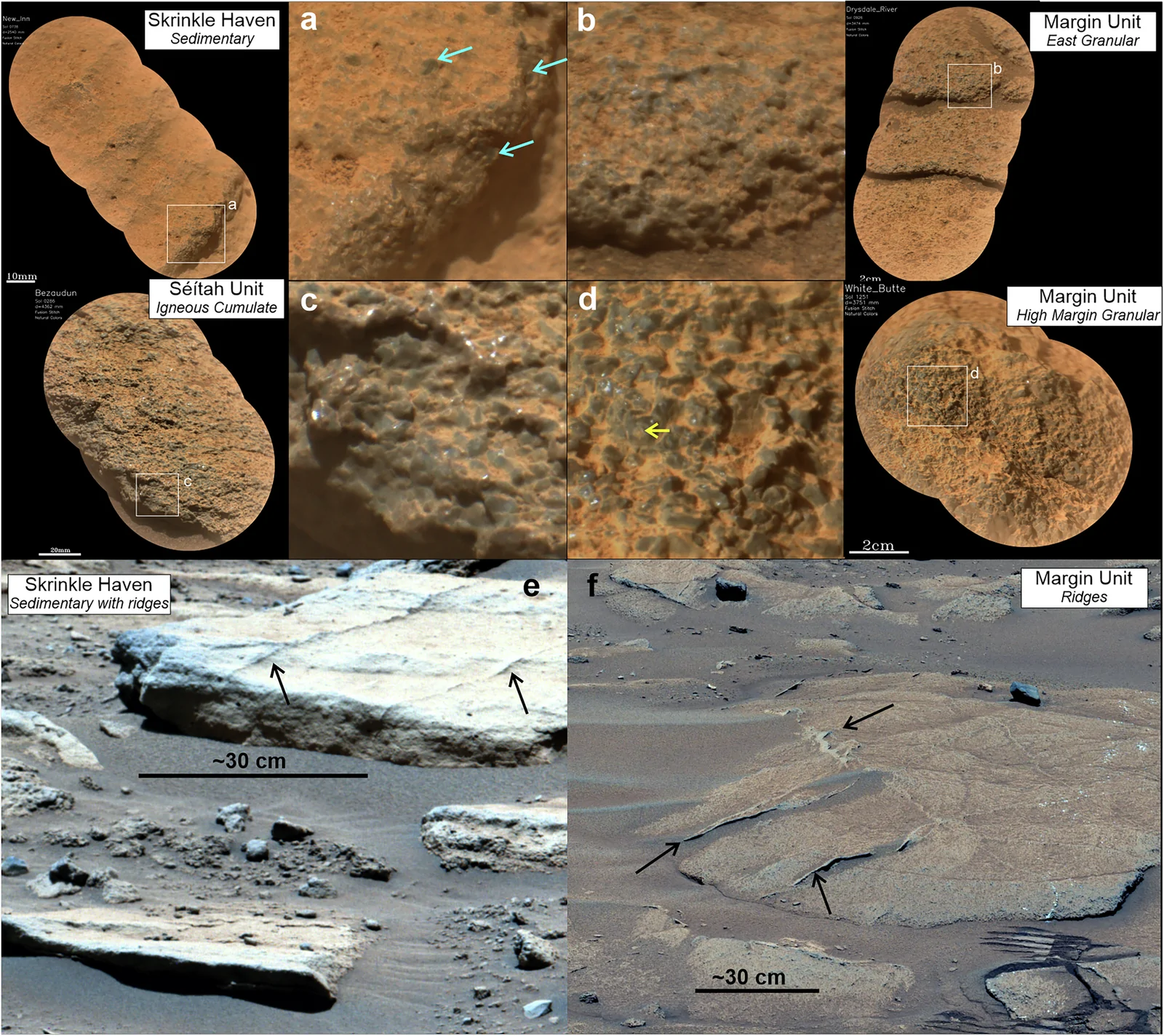
Comparison images between the sedimentary Skrinkle Haven mbr. of the Western Fan and the cumulative textures of the Séitah crater floor unit to the Margin unit. **a–d** are all 2 cm across to show a closer comparative view. Blue arrows in a) point to rounded, dark-toned grains in the sedimentary unit. The yellow arrow in (**d**) points to a lighter grain enclosed within a darker grain. **e** shows an enhanced color Mastcam-Z image mosaic of ridges within the Western fan Skrinkle Haven mbr (sol 750, ZCAM03591) and **f** shows an enhanced color Mastcam-Z image mosaic ridges in outcrop scale in the Eastern Margin (sol 932, ZCAM08941). Black arrows point to ridge features.

SuperCam data show that High Margin granular textures are indicative of a crystalline, in-place igneous unit. The subhedral to euhedral, abutting to interlocking grain shapes, equant grains, and thin interstitial material likely formed due to slow cooling and the accumulation of olivine in a magma body consistent with the interpretation by Williford et al^8^. These High Margin textures are also similar to textures observed in the olivine cumulate rocks of the Séitah formation of the Jezero crater floor^18,19^ (Fig. 7). Candidate poikilitic textures are observed in some RMIs (Fig. 7D) with lighter green chadacrysts enclosed in a darker colored grain. PIXL element maps of the Dourbes abrasion patch in Séitah also revealed a poikilitic texture with olivine enclosed by augite^18^, which supported an intrusive igneous, basal lava flow, or slow-cooling impact melt origin. Our results of the High Margin granular texture show a dominance of the olivine-rich cluster (Fig. 3B) with minimal contributions of carbonate clusters, supporting little overprint of the original texture by aqueous alteration processes^8,10,15^.

Some differences exist between Séitah and the High Margin. Mineralogically, Séitah has high and low calcium pyroxene alongside olivine^18,19^, whereas the High Margin has olivine with only low calcium pyroxene^15,20^. SuperCam chemical data also show that the Margin unit has greater MgO abundances and lower CaO abundances than Séitah^15^. These results could be consistent with differentiation and fractional crystallization within a layered intrusion, suggesting that these units may have occurred in a single or related magmatic events^8^.

The alteration extent of the East and West Margin complicates the determination of origin based on textures (e.g., Fig. 3D and Ravanis et al.^10^). However, SuperCam analyzed some targets representing the original crystalline texture, which were not investigated by the proximity science instruments. The West Margin granular texture displays the same grain shapes, abutting relationships to surrounding grains, grain size and composition as the High Margin Granular texture (Fig. 4 and S14). The Rubbly texture observed in the East and West Margin also displays similar features of crystalline, abutting grains with subhedral shapes in large grains that are actively eroding from recessive bedrock and shares an olivine-rich composition with minimal alteration (Fig. 3A). These textural classes suggest a continuation of the coarsely crystalline, olivine-rich igneous unit into the East and West Margin. Based on the lack of apparent grain rounding for the composite grains, it is unlikely that they occurred as pebbles transported in a river or lake shore environment such as the pebble-bearing beds of the Western Fan (Fig. 7). Instead, the Rubbly texture may have formed through relatively recent physical erosion of a less altered part of the Margin unit, potentially by eolian processes or thermal stress weathering.

Interestingly, the mean Mg# of the Rubbly texture olivine-rich cluster (52.7 ± 2.4) is lower than that of the High Margin and is more similar to the Mg# of the East Margin granular texture (54.1 ± 4.3), consistent with other studies showing a lower Mg# for the East Margin relative to the High Margin^8,15^. The presence of this lower Mg# in the Rubbly textural class with minimal secondary alteration suggests that this is inherent to the original bedrock and is not a factor of alteration. The RIMFAX Ground Penetrating Radar instrument onboard *Perseverance* detected basinward dipping reflectors indicative of thick layers throughout the Margin unit^8^. Layered intrusions crystallize from the contact with the country rock to the center of the intrusion, suggesting that the Margin unit reflectors closest to the crater rim are likely the oldest and therefore crystallized first in the succession. Therefore, this change in Mg#s, indicative of more forsteritic compositions in the High Margin and lower Mg#s in the East Margin, would be expected in a fractional crystallization scenario.

Some textures in the East Margin could be indicative of the transportation and deposition of an olivine-rich, crystalline source by lake-shore processes. Several East Margin Granular targets close to Turquoise Bay contain planar to wavy layering, some of which appear normally graded (Fig. S12). Measured grain sizes of the East Margin also overlap with those of the sedimentary, olivine-rich Skrinkle Haven mbr. of the Western fan (Fig. 4), though large, round, dark grains are absent (Fig. 7B). Olivine grains are found to be susceptible to rounding by physical abrasion^21^, and reworking of local olivine-rich bedrock would increase grain roundness and porosity that would enable the precipitation of a higher abundance of carbonate and silica as cement, similar to what has been seen in the Upper Jezero fan units^22^. Both PIXL and SuperCam show that the chemistry and mineralogy of the Upper Fan units are comparable to those of the Margin unit, including the secondary mineral assemblage with Fe/Mg carbonate, silica, and phyllosilicates^15,20^. Cross-bedding, convex-up stratification, erosion surfaces, and large-scale stratigraphic architectures of basinward and rimward dipping strata have also been identified in the East Margin, indicative of possible lake shore processes^9^. The occurrence of these candidate sedimentary features in the Margin unit closest to the Western Jezero fan (Fig. 1) may support this interpretation, as this is where wind-driven wave action in the Jezero paleolake is most likely to have eroded and reworked the local bedrock material^9^. However, all potentially sedimentary features relating to layering, cross-bedding, laminations and grading within layers can occur in layered igneous intrusions due to grain settling and convection-driven magma currents occurring within the magma body^23^. Furthermore, strong homogeneity of Ti/Cr ratios of chromite grains investigated by PIXL in the abrasion patches whose ratios are distinct between the East, West, and High Margin would suggest that any reworking of the Margin unit must have been at a very local level to preserve the distinct chromite populations between localities^8^.

**Fig. 8:**
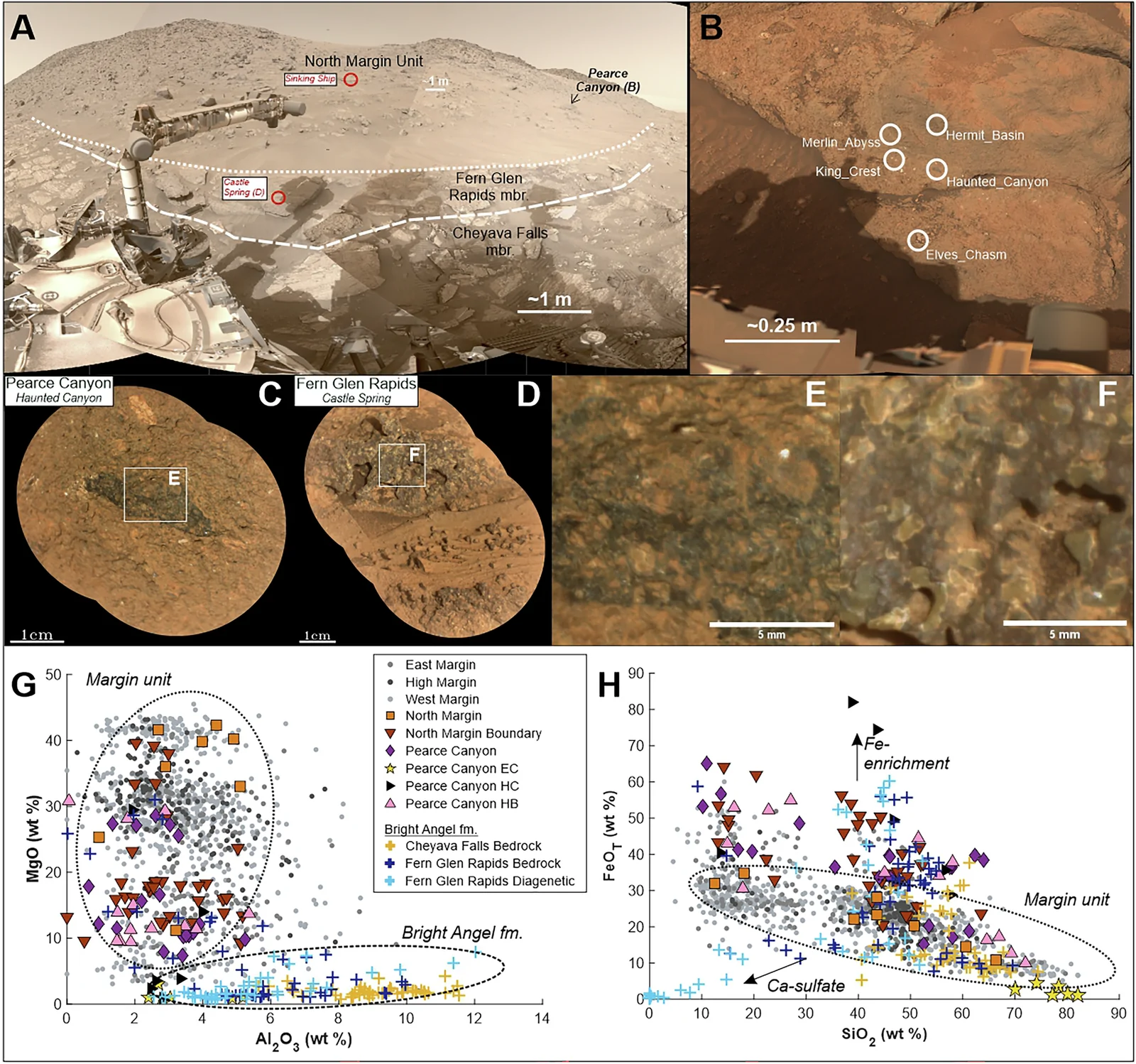
Compositional and textural comparison of the Pearce Canyon-type bedrock, North Margin unit rocks, and rocks of the Bright Angel Formation in Neretva Vallis with context images. **A** shows a Navcam mosaic (sol 1192) with boundaries between the North Margin unit and Fern Glen Rapids mbr. shown as a dotted line. The boundary between the Fern Glen Rapids mbr. and Cheyava Falls mbr. is shown as a dashed line. Red circles show targets of interest, and the arrow points to the location of Pearce Canyon. **B** shows a Navcam Left image (sol 1206, NCAM00709) of the Pearce Canyon outcrop with SuperCam target locations for Haunted Canyon (shown in **C**), Elves Chasm (shown in Fig. 3E), and others, shown as white circles. **C**, **D** show fused RMI mosaics of the Pearce Canyon target Haunted Canyon and the Fern Glen Rapids member target Castle Spring, respectively. **E**, **F** are close-ups of the Fe-rich, dark-toned coatings/matrix surrounding green grains in both the Pearce Canyon outcrop and Fern Glen Rapids member. **G**, **H** are biplots showing compositional differences between the East, West and High Margin (gray dots), North Margin (target Sinking_Ship acquired several meters above the boundary), North Margin boundary targets, Pearce Canyon matrix targets, Pearce Canyon Elves_Chasm clast (EC), Merlin Abyss (MA) target (top of outcrop), and Pearce Canyon Haunted Canyon target (HA, shown in **C**, **E**). Bright Angel FM. Members Cheyava Falls and Fern Glen Rapids bedrock compositions are also shown, as are compositions of diagenetic features in Fern Glen Rapids.

Sedimentary reworking of the Margin unit is evident at the boundary of the North Margin unit and Bright Angel fm. in Neretva Vallis (Fig. 1). The poor sorting, lack of structure or grain orientation, polymict, angular to subrounded clasts, and matrix-supported nature of the Pearce Canyon outcrop (Fig. 8) are indicative of a debris flow deposit^24^. SuperCam targeted a large, light-toned, rounded clast (Fig. 3E) which was found to be silica-rich and have an IR spectrum indicative of opaline silica^25^. Mineralogical diversity was also seen in a light-toned patch at target King Crest, where kaolin is detected (Fig. S15), but all other targets sampled the matrix material and a dark-toned patch. Pearce Canyon and targets close to the boundary with Bright Angel also exhibit FeO_T_ up to 60 wt% (Fig. 3V), uncommon to the rest of the Margin unit. High FeO_T_ is particularly prevalent in the dark-toned patch of the Haunted Canyon target (Fig. 8) with abundances up to 82 wt%. The Fern Glen Rapids member of the Bright Angel Fm. is an olivine-rich, coarse-grained, matrix-supported deposit situated closest to the boundary with the Margin unit (Fig. 8D) and is also interpreted as a debris flow deposit into a small lake within the Neretva Vallis channel^12^. In addition to coarse, angular, olivine grains, the Fern Glen Rapids member has several dark-toned diagenetic features with high FeO_T_ (Fig. 8D) and is situated above an Al-phyllosilicate-bearing unit^11,12^. Similar textures indicative of a debris flow depositional environment and similar Fe-enrichment associated with dark features suggest that the Pearce Canyon-type bedrock is more related to the sedimentary facies of Bright Angel and Neretva Vallis than the Margin unit. Although overlap exists, compositional differences for MgO and Al_2_O_3_ between the Pearce Canyon-type bedrock and the Fern Glen Rapids member suggest that the latter has a greater contribution of non-Margin unit material. Prior to these results, it was uncertain whether the Bright Angel Formation was younger than the Margin unit. However, the incorporation of olivine into these debris flow deposits associated with the Bright Angel Formation suggests that the Margin unit likely existed before.

### Groundwater or lacustrine alteration of the Margin unit?

A fluvial or lacustrine origin of Margin unit carbonates was hypothesized based on deep 2.5 µm absorption and low 2.3/2.5 µm band depth ratio in the orbital spectral data associated with the maximum estimated height of the Jezero paleolake^7^. However, later work shows that areas of the regional olivine-bearing unit outside Jezero crater exhibit similar carbonate spectral signatures to the Margin unit, suggesting hydrothermal or groundwater solutions were responsible for the alteration here and elsewhere in the region^26^.

Our results show that the Margin unit has textures and features indicative of variable and diverse aqueous alteration overprint across the unit (Figs. 3, 5–7; see also Ravanis et al.^10^). These alteration features are concentrated in certain areas of the East and West Margin at elevations below the estimated maximum lake height (Fig. 1), suggesting that interaction with the Jezero paleolake may have formed some of these features. However, their non-uniform spatial distribution below the lake level (Fig. 1) and the different chemical compositions could also support the influx of different groundwater systems, variably altering the bedrock.

**Fig. 9:**
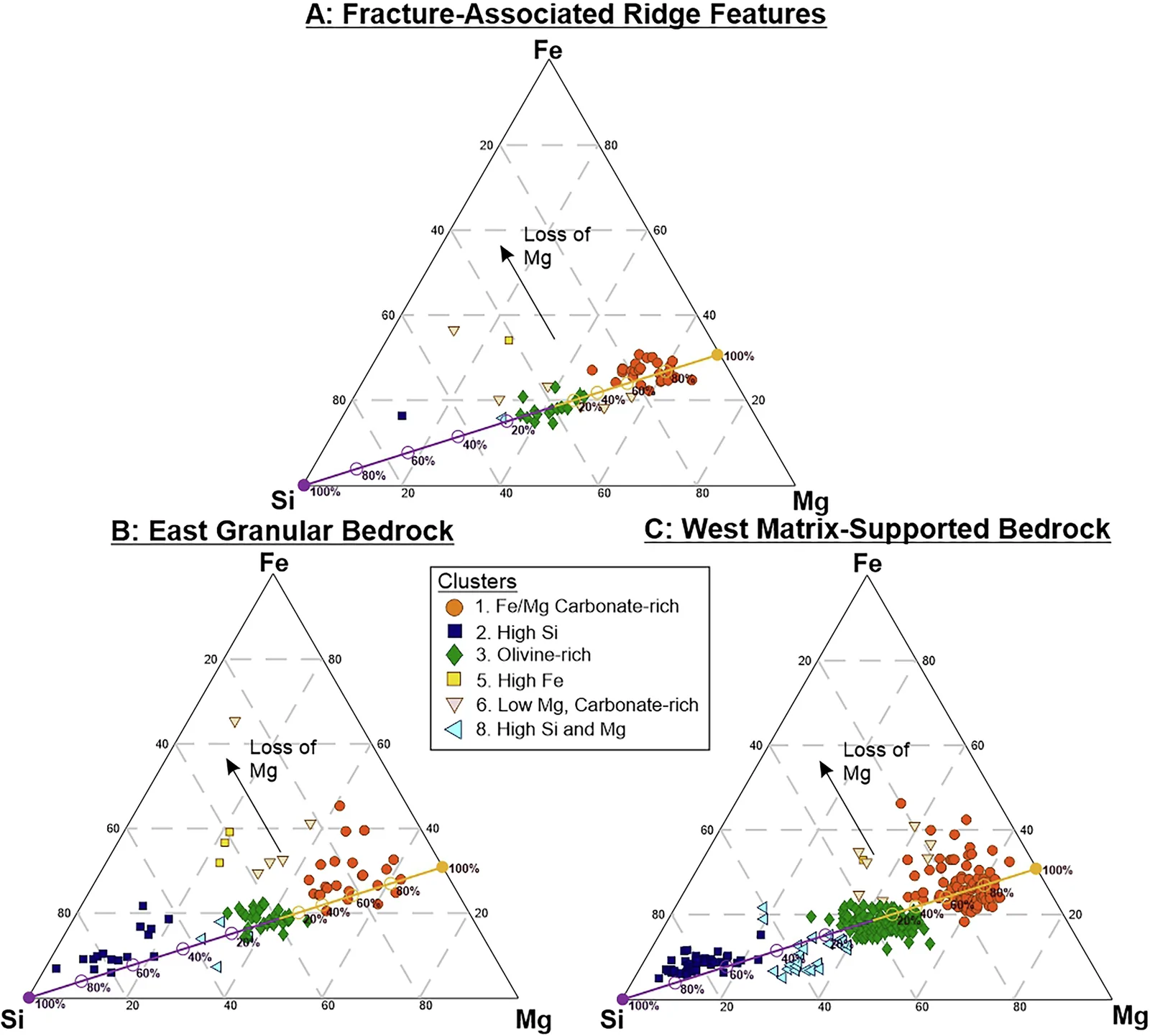
Ternary plots of Si, Fe, and Mg showing the compositions of each SuperCam Margin unit cluster in comparison to modeled compositions of carbonate (orange line) and silica (purple line). Carbonate compositions were modeled assuming the Fe/Mg of the original olivine is maintained (orange line). Numbers on the lines represent mixing between these carbonate and high silica modeled endmembers. Any deviation towards the Fe apex along this line is indicative of Mg remobilization from each phase. **A** shows the cluster compositions for fracture associated alteration features (ridges and concentric features), **B** shows the cluster compositions for East Margin Granular bedrock and **C** shows the cluster compositions for the West Margin Matrix-supported bedrock.

Ridges have been observed at many locations on Mars and are interpreted as erosion-resistant fracture fill relating to the migration of fluids through the bedrock^27–29^. Cluster results of both ridge types show a high percentage of carbonate-related cluster points ( > 50%) relative to the bedrock and have negligible high SiO_2_, suggesting that these features are carbonate-rich and were likely associated with the early carbonation of the Margin unit. This interpretation is consistent with uniform ridge carbonate compositions from the main Fe/Mg Carbonate-rich cluster 1 plotting with an equivalent Fe/Mg ratio to olivine (Fig. 9). Based on the more pronounced appearance of type 2 ridges, we interpret them to have formed from repeated periods of fluid circulation or greater precipitation of carbonate within the bedrock.

For the more altered bedrock of the East and West Margin unit, the cluster analysis reveals that silica is also a notable contributor and typically associated with the dark gray areas of the Matrix-supported textural class (Fig. 3D). PIXL and SHERLOC data support that silica is largely found as the matrix material surrounding altered olivine grains in the East and West Margin^8^. The solubility of silica and carbonates reaches maxima under different fluid conditions, with silica solubility enhanced by increasing temperature and pH and decreasing with elevated CO_2_; meanwhile, the reverse is true for carbonate solubility^30^. For example, quartz-carbonate terrestrial rocks called listvenites can become silica-dominated when circulating fluids become cooler and more acidic, favoring carbonate dissolution^30^. Earth-based studies into groundwater systems show that the combined effects of evaporation, cooling, and CO_2_-loss from a fluid increase silica saturation and its precipitation over carbonate^31,32^.

**Fig. 10:**
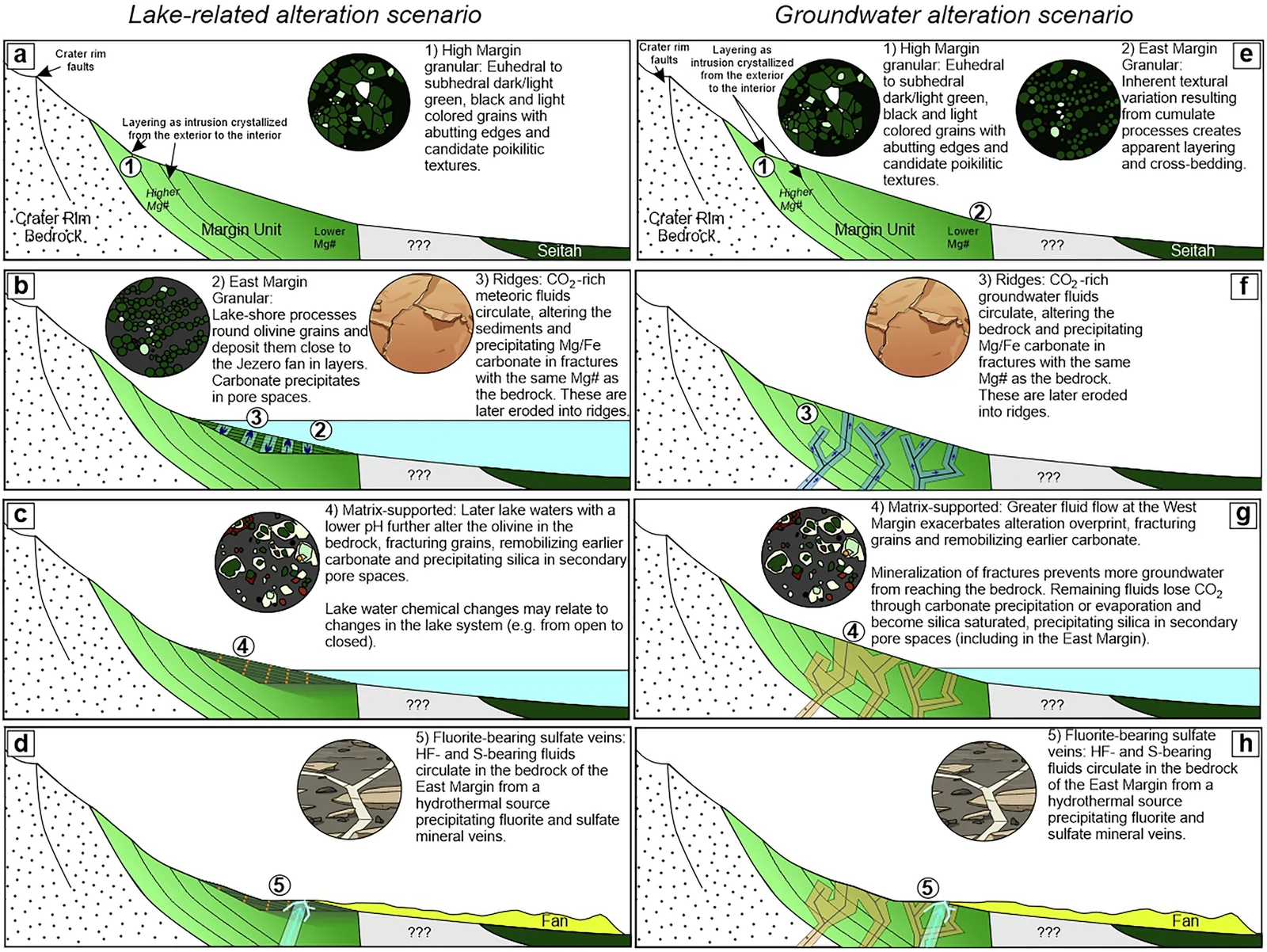
Schematic diagram of Margin unit formation and alteration events. Two endmember scenarios are presented following the layered intrusion emplacement model of Williford et al.^8^ depending on whether alteration was driven by lake–groundwater interactions or groundwater alone, and whether igneous cumulate or lakeshore sedimentation processes dominate. **A–C** Lake-related scenario: (1) Formation of the High Margin texture through crystallization of olivine within a possibly intrusive, layered, cumulate that was subsequently eroded and exposed. Layering is oriented according to the proximity to the border with the country rock (crater rim), with higher Mg# occurring closest to the boundary where crystallization initiated. (2) and (3) Reworking of the Margin unit by lakeshore processes rounds grains, reduces grain size, and creates pore space for carbonate precipitation. Stage 3 involves the early diagenetic ridge formation as CO_2_-rich fluids from the lake circulated within the olivine-rich material, carbonating it, including some detrital grains, and forming preferentially cemented, erosion-resistant linear features with the same Mg# as the olivine from the bedrock. (4) Further alteration forms the Matrix-supported texture as lower-pH fluids remobilize Mg from the carbonate, generate secondary porosity, and precipitate silica in the pore spaces while olivine grains become more altered and fractured. Lake pH changes may reflect changes in groundwater or meteoric water input, changes in climate, or lake evaporation later in its history^77^. **E–G** Groundwater-only scenario: (1,2) Formation of the olivine cumulate lithology, with textural variations in layering and grain shape driven primarily by magmatic cumulate processes. (3) CO_2_-rich groundwater circulates through bedrock fractures, precipitating carbonate with the same Mg# as the bedrock. (4) Greater groundwater flux in the West Margin drives enhanced alteration; as fractures become fully mineralized, remaining fluids become trapped and reach silica saturation as CO_2_ is lost through degassing or carbonate precipitation. **D**,**H** Late-stage alteration (both scenarios): (5) Formation of the fluorite-bearing sulfate veins, likely representing the latest stage of aqueous alteration.

One possibility for the Margin unit is that warm, CO_2_-rich groundwater fluids circulated in the fractures and saturated the Margin unit bedrock, altering the silicate minerals with Fe/Mg carbonates and eventually precipitating Fe/Mg carbonates in the fractures (Fig. 10). This made this carbonate-rich fracture fill more resilient to later erosion than the bedrock, forming ridges. Once the fractures were filled, the remaining fluid trapped within the bedrock cooled and evaporated, enabling the precipitation of silica in the remaining pore space. While this scenario describes a relatively closed system, the East and West Margin bedrock exhibits compositional scatter towards the Fe apex, away from the Fe/Mg ratios of the olivine compositions (Fig. 9), suggesting remobilization of Mg from the carbonate-rich bedrock component. Pitting is present in some Matrix-supported textures in the West Margin (e.g., Fig. S16), in addition to fractured grains (Fig. 3D), which could be indicative of secondary porosity and reaction-driven fracturing enhancing the alteration extent. This suggests that the later fluids also altered the carbonate, created secondary porosity, and precipitated the silica in the secondary pore spaces. No silica-rich veins have been observed in the Margin unit to suggest that the cooler fluids infiltrated in a later hydrothermal setting.

Based on the presence of the Western and Northern Jezero fan plus inlet and outlet fluvial channels, it is understood that Jezero crater contained closed- and open-system lakes that experienced several breaches of the eastern rim^33–36^. As such, it is possible that the lower temperature, lower pH fluids which saturated the bedrock may have been from the Jezero paleolake, preferentially altering the pre-existing carbonates therein (e.g., Fig. 10). Notably, the high silica cluster is only present in the East and West Margin which are below the second terrace level identified from orbital analysis as the lake level after a second breach event^33^ (Fig. 1). The sedimentary units of the Upper Western fan associated with deposition into the Jezero paleolake also contain high abundances of carbonate and silica^15,20^ and occur as secondary cement alongside some sulfate cement^22^ possibly supporting a shared alteration history.

Interestingly, the Séitah formation situated at the base of the crater has significantly less alteration compared to the East and West Margin or the Western fan^15^, which is unexpected if interaction with the Jezero paleolake waters is what caused this alteration across all units below the lake level. Previous studies hypothesized that Séitah may have been sufficiently protected from direct exposure to the lake waters by overlying units that have since been eroded away over time^10,37^. However, Séitah does not contain ridge features, unlike the Margin unit, suggesting that there was less fracture-related permeability when groundwater systems were active, which would have inhibited the water-rock interaction in Séitah relative to the Margin unit.

### Late-stage alteration of the Margin unit

Concentric features occur alongside the polygonal fractures that crosscut the ridges, suggesting that they formed later in the Margin unit’s history. Meter-scale concentric features seen in the East Margin contain greater abundances of carbonate closer to the edge of the feature compared to the core of the feature (Fig. 6), indicative of spheroidal weathering where chemical alteration preferentially occurs inwards from pre-existing joints or fractures^38,39^. Corners are preferentially altered due to the higher surface area, leaving a spherical, less altered core. Volume expansion during alteration creates micro-cracks that eventually separate the shells from the corestone^38^.

The Point Cloates target (Fig. 5C) is a ~ 25 cm thick mineral vein in the East Margin that stretches several meters to the east and northeast. The LIBS observations show that the target contains Ca-sulfate and CaF_2_ indicative of fluorite (Fig. 5). Given the low abundances of CaO, S, and F in the surrounding Margin unit bedrock, these fluids must have a different source to those that formed the ridges, potentially interacting with the diverse lithologies from the crater rim or the Ca-sulfate-bearing mudstones in Neretva Vallis^12^. Fluorine can also be enriched in Martian groundwater systems through the dissolution of F-bearing phases such as fluorapatite^40,41^. Typically, Ca-sulfates in Jezero crater are seen in cm- to mm-scale veins, as crystals or in patches, suggesting that they occurred as late-stage diagenetic features^12,22,42^. As such, it is likely that the vein also formed from a late-stage episode of aqueous alteration; however, the presence of CaF_2_ in the vein has different implications from the sulfates present in the bedrock within the area. Fluorite can precipitate as a minor phase in bedrock at low fluid temperatures ( < 13 °C) on Mars^41,43^, but the context of this occurrence in a large mineral vein is more similar to fluorite precipitation in hydrothermal settings on the Earth^44–46^. Indeed, fluorite mineral veins have been detected in Gale Crater alongside Ca-sulfate and Mg-rich diagenetic features^47^ but given the lack of volcanic rocks or features in the area, the formation scenario for these features remains unknown. In Jezero crater, the presence of a candidate volcanic edifice on the edge of Jezero crater^48^, igneous units of the crater floor, and Jezero crater’s proximity to the Syrtis Mons volcanic plateau suggest that later volcanism-induced hydrothermal circulation is a likely formation mechanism for fluorite vein precipitation at this site (Fig. 10).

## Conclusions

Our results demonstrate that the texture and chemistry of the Margin unit explored at high elevations are indicative of a slowly cooled, crystalline, olivine-rich ultramafic body. Near the Jezero Western fan, the East Margin exhibits clear indicators of sedimentary reworking, while the base of the North Margin unit shows evidence of polymict debris-flow deposits. Following emplacement, the Margin unit experienced at least three episodes of alteration: 1) neutral-to-alkaline, CO_2_-rich meteoric fluids formed carbonate-rich fracture fill that later eroded into ridges; 2) lower-pH solutions remobilized carbonate, driving secondary porosity generation and silica precipitation from cooler and/or silica-saturated fluids; and 3) late-stage hydrothermal fluids flowed through younger fractures, precipitating Ca-sulfate-rich and fluorite-bearing mineral veins. Given the lack of discrete silica veins, the fluids responsible for intergranular silica precipitation were likely supplied either by the Jezero paleolake itself or by groundwater evolving through sustained water–rock interaction. Overall, the Margin unit recorded a complex aqueous history driven by multiple alteration events from distinct groundwaters and/or exposure to the lake, cementing the Margin unit and the samples collected by *Perseverance* as primary targets of astrobiological interest.

## Methods

### SuperCam instrument overview

The SuperCam instrument suite onboard the Perseverance rover is situated 2 m above the surface on the rover mast and contains a telescope, 110 mm in diameter, which both projects laser beams and collects light from its geological targets^13,14^. The SuperCam instrument suite can acquire elemental data using Laser-Induced Breakdown Spectroscopy (LIBS), mineralogical data using Raman spectroscopy and Visible Near-Infrared Spectroscopy (VISIR), sound waves using a microphone, and high-resolution, color-context images of targets using the Remote Micro-Imager (RMI)^13,14^. This study characterizes the textures of SuperCam targets through analysis of RMI images and uses LIBS elemental data to estimate relative abundances of primary igneous minerals and secondary alteration minerals across different locations and textural classes.

The RMI is a three-color imager with a small field of view (18.8 mrad) whose purpose is to document the analytical area of the spectroscopy instruments^14^. The RMI has a focus range of 1.2 m – infinity, so can also be used to take high-resolution images of long-distance targets outside of the rover arm workspace^14,49^. Color images are acquired by using Bayer filters with a resolution ≤ 80 µrad, permitting the observation of 160 µm grains (fine sand) at 2 m distance^13^. RMI images are produced in either natural color, which is obtained through refining the red, green, and blue intensities relative to the white target on the rover deck^50^, or as a Gaussian stretch. The Gaussian stretch is created through independently stretching each color channel in the image so that the intensity distribution of the pixels for each color layer follows the same Gaussian distribution^51^. The initial characterization of the textural groups was performed in natural stretch; however, the Gaussian stretch better highlights grain boundaries and was thus used in the grain size distribution analysis. Due to the small field of view, several RMI images are acquired for a single geological target and stitched to form a mosaic.

The SuperCam LIBS uses a high-powered (14 mJ on target), pulsed, 1064 nm laser to ablate material from a small analytical area ( ~ 250 – 400 µm) on a geological target^13,14^ and excite it into a plasma. The plasma consists of atoms, ions, and molecules from the sample, which then, as the plasma cools and the atoms return to their neutral energy state, release photons that relate to the sample’s elemental composition^14,52^. The formation of this plasma also generates a shock wave, which has proven useful for clearing away dust from Martian rocks^14^. SuperCam LIBS data are typically acquired in a line or grid raster configuration (10×1, 5×1, or 3×3) with 30 laser shots per point in the raster for bedrock targets. Due to the presence of dust on the Martian surface, the first 5 laser shots are removed from the LIBS data to exclude the influence of dust. LIBS spectra are preprocessed to remove ambient light, noise, and the continuum signal, as well as to correct for signal fluctuations and target distance^53^. Calibration models are applied to preprocessed LIBS spectra to quantify elemental abundances for SiO_2_, TiO_2_, Al_2_O_3_, FeO, MgO, CaO, Na_2_O, and K_2_O for the average of each laser point in the raster^53^. The total sum of oxides is also provided by the models, whereby a decrease in totals for a target in focus is usually attributed to the presence of a non-quantified element, such as sulfur in a sulfate mineral vein or C in a carbonate. In this study, we use both Major-element Oxide Compositions (MOC) from the multivariate calibration models and scores generated by Independent Components Analysis (ICA) following the method of^54^. This has the benefit of adding elements not normally quantified by MOC, such as C, H, Li, and O to our investigation of relative elemental abundance and compares the results of the calibration models to the spectral data to check for any inaccuracies introduced by the models^55^. For instance, the current version of the MOC overestimates total abundances when FeO_T_ and MgO abundances are high^56^, so in the case of detecting Fe/Mg carbonates, we also rely on seeing elevated C scores from the ICA models not clearly associated with differences in distance (e.g., Fig. S2, 3). We also rely on detections of carbonates from other SuperCam techniques, such as the 2.3 µm and 2.5 µm bands in VISIR (e.g., Fig. S9) and carbonate detections made with Raman. In this study, we use LIBS targets observed between 2 and 6.5 m from the rover mast, as this is the range for which LIBS data has been quantified. Limited exceptions are made for targets of geological significance, such as the Point Cloates target that was taken at 7.8 m and the Victor Island ridge target taken at 7.0 m. Targets with poor focus were also excluded from the analysis.

### Image analysis

Three types of image analyses were undertaken in this study for each SuperCam target. First, Navcam (Navigation Camera) and Mastcam-Z (Mast Camera Zoom) rover images were investigated to determine the type of geological material targeted by SuperCam (e.g., regolith, float rock, alteration feature, in-place bedrock). Navcam consists of two engineering cameras with a 96° x 73° field of view and a resolution of 0.33 mrad/pixel that acquire color stereo images of the rover workspace for target selection^57^. The Mastcam-Z instrument is a higher resolution pair of cameras that provide stereoscopic, color zoom imaging with a field of view of 25.6° x 19.2° to 6.2° x 4.6° and can resolve features ~0.7 mm in size at 2 m distance from the rover^58^.

Due to the regolith-covered and fractured nature of the Margin unit, it became difficult to distinguish between bedrock observations and locally derived float at the small scale. As such, we created a size limiter so any target outcropping from the regolith that was within the size range of 2 cm – 15 cm across was automatically placed into a float rock category. Regolith targets include any SuperCam target of unconsolidated sedimentary material that is finer than 2 cm in size (granule grain size in the Wentworth scale). If the target is larger than 15 cm and has textural characteristics of the surrounding bedrock, then it is considered a bedrock target. Alteration features are classified as textures within the bedrock that differ in color, apparent roughness, and surface relief, such as veins, ridges, or concretions. These features are placed into a separate category from the bulk bedrock observations as they likely relate to later episodes of water-rock interactions. Other targets not included in the Margin unit bedrock dataset include those taken from rocks within the channel that runs along the eastern edge of the Margin Unit or within Neretva Vallis that are chemically and mineralogically distinct from the Margin unit itself.

Once the SuperCam bedrock targets were identified, they were further subdivided according to their broad textural features in RMI images such as grain shape, grain relationships (matrix- or grain-supported), and rock fabric (e.g., the presence or absence of layering/laminations). Grain shape was determined qualitatively, and a matrix was defined as interstitial material that has an unresolvable grain size. Alteration features were also subdivided according to their main morphologies and similarity in appearance to the surrounding bedrock. If textures were obscured by poor focus, dust cover, or poor lighting conditions, the targets were classed as “unknown”. Once classed according to their textures, the SuperCam bedrock and alteration feature targets were then mapped across the Margin unit using the Campaign Analysis Mapping and Planning (CAMP) GIS tool^59,60^ to constrain their spatial distribution and relationship to elevation.

Finally, RMI image mosaics with good focus and visibility of the bedrock textures were selected for grain size analysis using ImageJ (https://imagej.nih.gov/ij/). Scales were determined according to the distance from SuperCam to the target and were imported into ImageJ using the Set Scale function. Then, the image was cropped to remove outer areas that may be distorted or unfocused, and a grid was generated as an overlay^61^. Using the polygon tool, visible grains at the grid intersections were measured. However, due to the weathered nature of the natural outcrop surface, discrete grains can be difficult to identify, and where a grain was not visible at the grid intersection, the closest grain to the intersection was measured instead. Similar to the methods of Wiens et al.^51^, the Gaussian stretch was used to facilitate the identification of grains based on color, and surface relief was also used as a guide to differentiate boundaries between grains. The polygon tool provides measurements of the long and short visible axes of grains, grain circumference, area, and an estimate of grain roundness. At least 150 grains were measured for each SuperCam target with clear RMI images of the outcrop (see Table S1 for the list of targets used).

### Cluster analysis

Due to the small analytical area of the SuperCam LIBS instrument ( ~ 250–400 µm), substantial scatter in the LIBS elemental compositions exists in the Margin unit dataset, reflecting a grain size close to or greater than that of the laser spot^62^. As such, the geochemical scatter is indicative of diverse mineral components of the bedrock, not a bulk composition, and a cluster analysis can be used to estimate relative mineral abundances across each textural class and location in the Margin unit. Cluster analysis has been successfully used in Gale Crater on ChemCam LIBS data in combination with an image-based analysis of sedimentary features to constrain mineral sorting with eolian transportation in the Stimson formation^41,63^. Cluster analysis of LIBS data has also been successfully applied to identify mineral components of regolith in Gale crater^64^ and detect chemostratigraphic changes relating to changes in major geological groups^65^.

Hierarchical agglomerative clustering is an unsupervised machine learning algorithm that starts with each data point as its own cluster and iteratively merges the closest clusters based on distance relationships until only one cluster remains. An unsupervised model was used to minimize human bias in the data analysis and account for the unknowns involved in Mars exploration, particularly given the large number of SuperCam targets encompassing a wide range of rock types not always analyzed by proximity science instruments due to engineering constraints (such as rock surface or size) or restrictions on rover resources (such as power or time). We use the *clusterdata* MATLAB® function from the R2022a Statistics and Machine Learning Toolbox^TM^ to perform a hierarchical agglomerative cluster analysis on the SuperCam Margin unit bedrock dataset. This dataset includes the MOC-derived abundances and ICA scores for each observation point included in the Margin unit bedrock dataset, which excludes regolith material and targets on small float that may not be locally derived.

To ensure equal weighting of each element, variables x with a mean of μ and standard deviation of σ were standardized with z scores using the *zscore* function:

$$z=\frac{(x-\mu )}{\sigma }$$

Data standardized in this way have a mean of 0 and a standard deviation of 1.

Next, the distance matrix was calculated using the *pdist* function. Several distance measures were tried for the cluster analysis, but the Manhattan (or cityblock) distance provided the best separation of clusters. Given a matrix *X* with row vectors *x*_*1*_*, x*_*2*_*,…, x*_*n*_, the distance between vectors *x*_*s*_ and *x*_*t*_ are calculated according to:

$${d}_{{st}}=\,{\sum }_{j=1}^{n}\left|{x}_{{sj}}-{x}_{{tj}}\right|$$

Based on the results of the *pdist* function, the *linkage* function was used to determine how objects are grouped into clusters. Similar to the methods of Bedford et al.^41^, complete linkage was used to cluster data which the MATLAB algorithm defines as:

$$d\left(r,s\right)=\max \left({dist}\left({x}_{{ri}},{x}_{{sj}}\right)\right),i\,\in \left(1,\ldots ,{n}_{r}\right),\,j\in (1,\ldots ,\,{n}_{s})\,$$

Where *r* and *s* are clusters, *x*_*ri*_ is the ith object in cluster *r*, *x*_*sj*_ is the jth object in cluster *s*, *n*_*r*_ is the number of objects in cluster *r*, and *n*_*s*_ is the number of objects in cluster s.

The cluster output is viewed as a dendrogram (Fig. S1) with similarity on the y-axis and variables on the x-axis. The dendrogram is used to identify the cutoff for the maximum number of clusters, and the silhouette score is used to determine the cohesion of the clusters. The number of clusters was determined according to a large change in Distance Levels (greater than 7) and by comparing the final cluster chemistry to mineralogy determined from proximity science instrument observations of abrasion patches^8^ and SuperCam VISIR and Raman data^15^.

Silhouette scores were used to test the similarity of data points in their assigned clusters to data points in other clusters and were calculated using the *silhouette* MATLAB® function from the R2022a Statistics and Machine Learning Toolbox^TM^. The formula for calculating silhouette scores for the *i*th point is below:

$${s}_{i}=\frac{({b}_{i}-{a}_{i})}{\max ({a}_{i},{b}_{i})}$$

Where the average distance from the data point *i* to the other points in the same cluster is given by *a*_*i*_, and *b*_*i*_ is the minimum average distance between the data point *i* and the data points in a different cluster.

Silhouette scores are calculated as a value between -1 and 1. Any data point with a silhouette score <0.2 is considered to have a poor relationship to its assigned cluster and is manually assigned a cluster membership of 0.

## Supplementary information

Transparent Peer Review file

Supplemental Material

## Data Availability

All Perseverance datasets used in this study are publicly available in the NASA Planetary Data System (PDS). SuperCam radiometrically calibrated RMI images (urn:nasa:pds:mars2020_supercam:data_radcal_rmi) and derived spectra (urn:nasa:pds:mars2020_supercam:data_derived_spectra) are hosted by the PDS Geosciences Node (DOI: 10.17189/1522646)^66^. Calibrated Mastcam-Z imagery (urn:nasa:pds:mars2020_mastcamz_ops_calibrated, DOI: 10.17189/bs6b-4782)^67^ and calibrated Navcam imagery (urn:nasa:pds:mars2020_navcam_ops_calibrated, DOI: 10.17189/yvkm-rx37)^68^ are hosted by the PDS Cartography and Imaging Sciences Node. SuperCam target classifications and cluster analysis output are available in the online repository^69^ and supplemental information in this manuscript.
