# Supplementary material for: Lake- and groundwater-associated alteration of the olivine-rich Margin unit in Jezero crater, Mars
Source: Commun Earth Environ. 2026 Sep 21;7(1):728. doi: 10.1038/s43247-026-03997-9 (PMC13593571; doi:10.1038/s43247-026-03997-9)
Supplement: Supplementary file 1 [file 43247_2026_3997_MOESM1_ESM.pdf]

nature portfolio

Peer Review File

Lake- and groundwater-associated alteration of the olivine-rich Margin unit in Jezero crater, Mars.

Corresponding Author: Dr Candice Bedford

This file contains all reviewer reports in order by version, followed by all author rebuttals in order by version.

Version 0:

Reviewer comments:

Reviewer #1

(Remarks to the Author)
Review of “Investigating the origin and lake-associated alteration of the olivine-rich Margin unit in Jezero crater, Mars” by Bedford et al.
Brian Hynek
University of Colorado
3/13/2026

This manuscript provides a detailed description of the various Margin sub-units traversed by the Perseverance rover in Jezero crater, Mars. This unit was identified prior to landing as carbonate-bearing and was thought to possibly represent a lake shoreline unit. The focus of this manuscript is on chemical and microscopic image data collected by the rover’s SuperCam instrument. The conclusions, based on petrography and chemical data are that the Margin unit was likely originally emplaced as an olivine-rich cumulate magma body. Evidence was shown for three distinct episodes of aqueous alteration. The multiple generations of water make this unit one of astrobiological interest.

Prior work (Jones et al., 2026) provided a stratgraphic analysis of the Margin unit and concluded that the western unit was likely a carbonated olivine cumulate and the eastern represented a beach environment that reworked materials from the western unit. The interpretation herein is similar, although with less focus on beach reworking and instead favoring a hypothesis that the eastern unit retains primary characteristics from the cumulate, like the western unit. After formation, data are presented that show three distinct episodes of aqueous alteration representative of varying fluid chemistry and sources.

The claims and geologic history reported within the paper are generally well-supported by the data. Particularly, the data supporting an olivine cumulative across the entire unit is very convincing. The lower reaches (east and west units) were altered by water multiple times. Convincing data were shown for the sequence of carbonization of the unit, then replacement by minerals formed from a lower pH fluid, and finally potential hydrothermal circulation to emplace Ca-sulfate veins. This seems like the most likely scenario from the data presented and I find no major flaws with the interpretation. In sum, the paper and evidence presented are scientifically sound.

There are lingering issues of minor importance or that cannot be answered with the current data. For example, lake fluid contributions compared with groundwater. The schematic shows a lake providing the carbonating fluids and then a lake with low pH fluids to remove it. It was not explained how this scenario is plausible. This also requires a long-lived lake of changing chemistry (or multiple lakes through time). Some additional discussion of the proposed scenario and any data to support it is welcome. Groundwater episodes are also provided as a possibility and in my opinion, much more likely. The crater rim nearby is heavily fractured and provides conduits. How can a lake not alter floor units like Seitah but pervasively alter the margin units? A brief idea was provided but was not terribly convincing.

Also, how does a cumulate magma body form on the side of a crater wall? This is perplexing. Did it extend across the entire crater? Probably not. Why is it here and not other places? This is very enigmatic so please provide at least some ideas to support a layered cumulate pasted on the side of a crater wall, which is not common on Mars. I am also not convinced of the possible laminations, or even the roundness of grains in Figure 3c. OK, there are a couple roundish grains visible but also a lot that are similar to Fig 3b, which is thought to be a cumulate. The possible laminations cannot be traced beyond the lines in Figure 3C. Do they just stop? Why? Collectively, I see little evidence to support any significant beach reworking of

deposits. The authors did not stress this idea, so perhaps it's ok as is. In my opinion, the beach reworking idea is tenuous as best from the presented data. Also, the "red vein" identified is challenging to see in the image and not discussed in the paper.

A few other minor comments:
1. You discuss low pH fluids. I would say "lower" or "relatively lower". It doesn't take too low of pH to dissolve carbonates.
2. Show the possibility of groundwater processes in your summary geologic history figure (not just the lake scenario).
3. Fluorite can form from evaporation of brines/marine waters, although I agree hydrothermal is more likely. Are there any chemical or observations that can distinguish these? It's an important issue. Also, Jezero is at the base of Syrtis mons and that could be a source of late stage hydrothermalism.

In summary, I feel the authors provided a convincing story for the history of the Margin unit, that represents a significant advancement in our current understanding of this enigmatic material. It is a strong contribution and should be published in Communications Earth and Environment with minor changes.

Reviewer #2

(Remarks to the Author)
This manuscript focused on the alteration of the olivine-rich Margin unit in Jezero crater, Mars by investigating data cluster Supercam LIBS analyses along with sed/strat and lithological considerations through image analysis using Navcam and Mastcam-Z, onboard the Perseverance rover in Jezero crater, Mars. The Nilli-Syrtis region has widespread olivine and carbonate formation on Mars and Jezero crater has an olivine-carbonate rich rocks, and a strong carbonate signature in the "Margin unit" that could be a prime location for the exploration of preserved biosignatures. The unsupervised modeling yielded 8 distinct clusters: 1) Mg-rich carbonate, 2) Si-rich phase, 3) Olivine, 4) Dust, 5) Fe-rich, 6) Low Mg, carbonate-rich, 7) Ca-sulfate with CaF & Mg-sulfate, 8) Mg silica-rich. Results from this study show that textures and chemistry of the Margin unit at higher elevations are slow cooled, crystalline, olivine-rich ultramafic body, and that it experienced at least 3 episodes of alteration throughout its history. These 3 episodes include: 1) neutral to alkaline CO_2-rich groundwater or lake water formed carbonate ridges, 2) carbonate remobilization by low pH and precipitation of silica into secondary pore space, 300 late-stage, sulfate-bearing fluids flowing through fractures and precipitating Ca-sulfate-rich veins.
• This manuscript was well written and is novel in the approach combining images and analyses to further our knowledge of the Margin unit and the alteration of sediments in Jezero crater. This work is important for understanding alteration on Mars and environments that could have potentially hosted life. Most of the comments are cosmetic with a couple of items requiring clarification and expanded upon.
• Lines 48-450: Lacked discussion on permeability and porosity that could potentially play a role in addition to the surface area aspect as a function of the small grain size could influence the degree of alteration of the olivine. It is unclear if the pore spaces were filled, or only partially filled with silica and this could have important implications for alteration. Along the same lines Line 48-9: Finer grain size "may have enabled greater alteration" . It is unclear what the specific size of "finer grain size" is meaning. For example, if the grain size is in the clay size fraction (<2um) then fluids precipitants would have a greater propensity to alter sediments horizontally/laterally more than vertically. This is where porosity and perm come in.
• Figure 1 "C": This figure is nicely laid out but challenging to distinguish dots other than the red ones. It was difficult to see the orange dots on the map in "C", and why are there so few orange "East and North Granular" compared to the red "High and West granular"? Are there 2 pink "Rubbly" dots? Dots are hard to see. Why are there more Mastcam-Z of some ridge targets and some Supercam of other ridge targets?
• Include N arrow on maps, and orbitally defined unit colors need a key of their own.
• Line 77- olivine-bearing rocks at Mast Peak (where is this on a map? Maybe I missed it).
• Line 106 and lines 633-635: The unsupervised model yielded 8 clusters there have been more/less if it was a supervised model vs. the unsupervised?
• Line 108- what is a "silhouette score"; please make sure you expand on the terminology, not everyone will know what this is.
• Figure 2 – are the error bars on this data less than the symbols?
• Lines 469-470; where is the fluorite coming from? What is the source of fluoride?
• Line 498 – what is the mechanism that is specifically responsible for the pH to drop?

Version 1:

Reviewer comments:

Reviewer #1

(Remarks to the Author)
My comments have been sufficiently addressed, and I think the authors did a fine job addressing the other referee's suggestions as well. I am mostly pleased with the state of the manuscript. There are some editing issues to address. Repeatedly in the text and also figure captions, the authors talk about the carbonate-rich fluids forming ridges (e.g., line 436, Figure caption 10, and beyond). The minerals were precipitated in fractures in the bedrock. Presumably, later differential erosion led to the harder materials to become manifest as ridges. This need to be clarified. Supplementary materials have an incomplete author list: "...Adrian Brown, Erwin Dehouck, anyone else? Katie Stack, Susanne Schroder?" There are other minor editorial issues throughout, so a thorough review by the authors is warranted.

Reviewer #2

(Remarks to the Author)
The manuscript edits are reasonable support the proposed major claims, and therefore I recommend publication of this manuscript.

Thank you

Open Access This Peer Review File is licensed under a Creative Commons Attribution 4.0 International License, which permits use, sharing, adaptation, distribution and reproduction in any medium or format, as long as you give appropriate credit to the original author(s) and the source, provide a link to the Creative Commons license, and indicate if changes were made.
In cases where reviewers are anonymous, credit should be given to 'Anonymous Referee' and the source.
The images or other third party material in this Peer Review File are included in the article's Creative Commons license, unless indicated otherwise in a credit line to the material. If material is not included in the article's Creative Commons license and your intended use is not permitted by statutory regulation or exceeds the permitted use, you will need to obtain permission directly from the copyright holder.
To view a copy of this license, visit https://creativecommons.org/licenses/by/4.0/

Response to reviewers:

Reviewer #1 (Remarks to the Author):

Review of “Investigating the origin and lake-associated alteration of the olivine-rich Margin unit in Jezero crater, Mars” by Bedford et al.
Brian Hynek
University of Colorado
3/13/2026

This manuscript provides a detailed description of the various Margin sub-units traversed by the Perseverance rover in Jezero crater, Mars. This unit was identified prior to landing as carbonate-bearing and was thought to possibly represent a lake shoreline unit. The focus of this manuscript is on chemical and microscopic image data collected by the rover’s SuperCam instrument. The conclusions, based on petrography and chemical data are that the Margin unit was likely originally emplaced as an olivine-rich cumulate magma body. Evidence was shown for three distinct episodes of aqueous alteration. The multiple generations of water make this unit one of astrobiological interest.

Prior work (Jones et al., 2026) provided a stratigraphic analysis of the Margin unit and concluded that the western unit was likely a carbonated olivine cumulate and the eastern represented a beach environment that reworked materials from the western unit. The interpretation herein is similar, although with less focus on beach reworking and instead favoring a hypothesis that the eastern unit retains primary characteristics from the cumulate, like the western unit. After formation, data are presented that show three distinct episodes of aqueous alteration representative of varying fluid chemistry and sources.

The claims and geologic history reported within the paper are generally well-supported by the data. Particularly, the data supporting an olivine cumulative across the entire unit is very convincing. The lower reaches (east and west units) were altered by water multiple times. Convincing data were shown for the sequence of carbonization of the unit, then replacement by minerals formed from a lower pH fluid, and finally potential hydrothermal circulation to emplace Ca-sulfate veins. This seems like the most likely scenario from the data presented and I find no major flaws with the interpretation. In sum, the paper and evidence presented are scientifically sound.

There are lingering issues of minor importance or that cannot be answered with the current

data. For example, lake fluid contributions compared with groundwater. The schematic shows a lake providing the carbonating fluids and then a lake with low pH fluids to remove it. It was not explained how this scenario is plausible. This also requires a long-lived lake of changing chemistry (or multiple lakes through time). Some additional discussion of the proposed scenario and any data to support it is welcome. Groundwater episodes are also provided as a possibility and in my opinion, much more likely. The crater rim nearby is heavily fractured and provides conduits. How can a lake not alter floor units like Seitah but pervasively alter the margin units? A brief idea was provided but was not terribly convincing.

We thank Dr Hynek for his recommendations relating to the groundwater alteration scenario and have worked to incorporate this into the text (see the Discussion section 3.2 and figure 10). We do believe it is a good addition and a groundwater only scenario would be a simpler way to explain the lack of alteration in Seitah compared to the Margin unit, and the occasional lenses of unaltered material in the East and West Margin in the Rubbly textural class. However, we would like to highlight that there is extensive geomorphological evidence that Jezero crater hosted open- and closed-system lakes (as discussed in lines 422-431) and many factors can cause lake water pH to change with time such as changes in meteoric water input, changes in climate, or evaporation rate. The similar carbonated grains, silica and sulfate cement seen in the sediments of the Western Jezero fan to those in the East and West Margin unit also suggests that this could have formed from shared lake water interactions and the lack of silica veins as fracture fill in the bedrock indicates that groundwater fluids were not solely responsible for the formation of silica so we keep the lake water alteration scenario as a possibility.

Also, how does a cumulate magma body form on the side of a crater wall? This is perplexing. Did it extend across the entire crater? Probably not. Why is it here and not other places? This is very enigmatic so please provide at least some ideas to support a layered cumulate pasted on the side of a crater wall, which is not common on Mars.

This is a great question and is an area of active research in the Mars 2020 team. For now, we would like to refer Dr Hynek to Williford et al. (2026), who discusses and introduces the cumulate geological model for these deposits (see their Fig. 7 for the hypothesized emplacement of this deposit relative to the rim). Our paper is more focused on relating the finer scale textures and their associated compositions/mineralogy observed with the diversity of SuperCam targets to the current geological models, in particular those from the ridges and mineral veins which were not analyzed by proximity science instruments but

which we believe play an important role in the alteration history of this unit. As such, we believe the emplacement mechanism for the cumulate body is out of scope of our paper and instead refer the reader to Williford et al. for more information in the text and Fig. 10 caption.

I am also not convinced of the possible laminations, or even the roundness of grains in Figure 3c. OK, there are a couple roundish grains visible but also a lot that are similar to Fig 3b, which is thought to be a cumulate. The possible laminations cannot be traced beyond the lines in Figure 3C. Do they just stop? Why? Collectively, I see little evidence to support any significant beach reworking of deposits. The authors did not stress this idea, so perhaps it's ok as is. In my opinion, the beach reworking idea is tenuous as best from the presented data. Also, the "red vein" identified is challenging to see in the image and not discussed in the paper.
We acknowledge that the possible laminations are qualitative and up for debate (hence why we refer to them as "possible" or "candidate" laminations in the text), but we believe we do see lineations present in this target, as well as in other RMIs from this area (e.g. see Fig. S12). So, these and the larger scale bedform features at this location discussed by Jones et al. and geomorphological features indicative of fluviolacustrine processes nearby makes us consider them worthy of a mention. But, as Dr Hynek mentions, we do not discuss them to a great extent. The lamination annotations are designed to not be continuous so readers can see for themselves the strength of the observations. We have removed mention of the "red vein" from the image annotations as recommended and have reduced the mentions of the higher abundance of round grains as they are harder to distinguish in the RMIs. However, rounder grains were also observed at this location in the Bills Bay abrasion by WATSON (see Jones et al. or Williford et al.) so we do consider our initial interpretation correct, but again it is a qualitative one as we did not measure grain shape.

A few other minor comments:
1. You discuss low pH fluids. I would say "lower" or "relatively lower". It doesn't take too low of pH to dissolve carbonates.

We have made the recommended changes to the text.
2. Show the possibility of groundwater processes in your summary geologic history figure (not just the lake scenario).

We have made the recommended changes to Fig. 10.
3. Fluorite can form from evaporation of brines/marine waters, although I agree hydrothermal is more likely. Are there any chemical or observations that can distinguish

these? It’s an important issue. Also, Jezero is at the base of Syrtis mons and that could be a source of late stage hydrothermalism.

We have added Syrtis Mons as another possible source of hydrothermalism and expanded the discussion on the fluorite mineralization. Reading the literature on Earth and Mars, fluorite precipitating from brines usually occurs within the bedrock, not in veins. Mineralization in veins appears to be mostly a hydrothermal setting. However, the fluorite-bearing sulfate veins in Gale crater have an uncertain origin, mostly as a high-temperature fluid origin is unknown here.

In summary, I feel the authors provided a convincing story for the history of the Margin unit, that represents a significant advancement in our current understanding of this enigmatic material. It is a strong contribution and should be published in Communications Earth and Environment with minor changes.

Thank you for your insightful review and we hope your find our modifications satisfactory.

Reviewer #2 (Remarks to the Author):

This manuscript focused on the alteration of the olivine-rich Margin unit in Jezero crater, Mars by investigating data cluster Supercam LIBS analyses along with sed/strat and lithological considerations through image analysis using Navcam and Mastcam-Z, onboard the Perseverance rover in Jezero crater, Mars. The Nilli-Syrtis region has widespread olivine and carbonate formation on Mars and Jezero crater has an olivine-carbonate rich rocks, and a strong carbonate signature in the “Margin unit” that could be a prime location for the exploration of preserved biosignatures. The unsupervised modeling yielded 8 distinct clusters: 1) Mg-rich carbonate, 2) Si-rich phase, 3) Olivine, 4) Dust, 5) Fe-rich, 6) Low Mg, carbonate-rich, 7) Ca-sulfate with CaF & Mg-sulfate, 8) Mg silica-rich. Results from this study show that textures and chemistry of the Margin unit at higher elevations are slow cooled, crystalline, olivine-rich ultramafic body, and that it experienced at least 3 episodes of alteration throughout its history. These 3 episodes include: 1) neutral to alkaline CO2-rich groundwater or lake water formed carbonate ridges, 2) carbonate remobilization by low pH and precipitation of silica into secondary pore space, late-stage, sulfate-bearing fluids flowing through fractures and precipitating Ca-sulfate-rich veins.

• This manuscript was well written and is novel in the approach combining images and analyses to further our knowledge of the Margin unit and the alteration of sediments in Jezero crater. This work is important for understanding alteration on Mars and environments that could have potentially hosted life. Most of the comments are cosmetic

with a couple of items requiring clarification and expanded upon.

• Lines 48-450: Lacked discussion on permeability and porosity that could potentially play a role in addition to the surface area aspect as a function of the small grain size could influence the degree of alteration of the olivine. It is unclear if the pore spaces were filled, or only partially filled with silica and this could have important implications for alteration.

We thank the reviewer for highlighting the benefit in a permeability/porosity discussion. We have tried to work in more discussion around this in lines 406-411 and 434-439, but it is difficult to confidently discuss the degree to which pore spaces were filled with each alteration event from rover images and with our remote science dataset, particularly due to the uncertainty around the degree of reworking the Margin unit experienced and given the alteration overprint it has experienced. We hope that we can return the collected samples to Earth one day so a detailed microscopy study can be conducted which can shed more light on this topic.

Along the same lines Line 48-9: Finer grain size “may have enabled greater alteration” . It is unclear what the specific size of “finer grain size” is meaning. For example, if the grain size is in the clay size fraction (<2um) then fluids precipitants would have a greater propensity to alter sediments horizontally/laterally more than vertically. This is where porosity and perm come in.

As mentioned in the Results section, the finest resolvable grain size in our images is 0.18 mm, so we cannot confidently determine whether the grains are fine enough to impact the direction in which the bedrock was altered or really expand more into a porosity/permeability discussion. As the study we cited does detail that the clay fraction is where the most notable difference in carbonation efficiency occurs, and given our uncertainties in measuring this grain size, we have removed this from the discussion, instead focusing more on the fracture-related permeability at the outcrop- and grain-scale which we can see in our remote science instrument images.

• Figure 1 “C”: This figure is nicely laid out but challenging to distinguish dots other than the red ones. It was difficult to see the orange dots on the map in “C”, and why are there so few orange “East and North Granular” compared to the red “High and West granular”? Are there 2 pink “Rubbly” dots? Dots are hard to see. Why are there more Mastcam-Z of some ridge targets and some Supercam of other ridge targets?

We have changed the size of the orange markers and Rubbly class markers in “C” and changed the color of the border to make them stand out more. The reason for the difference in number relates to our ability to clearly distinguish texture when imaging the

natural surface. As mentioned in the Methods section, there are some targets that texturally were obscured by substantial dust or coating coverage. Unfortunately, the East Margin unit was particularly prone to being covered in dust so there are fewer confident detections of this textural class here and the other targets have been labelled as “unknown”. We have tried to clarify this in the Methods section and the Figure caption.

For the Mastcam-Z vs SuperCam ridge detections, this relates to rover resources and tactical priorities. Mastcam-Z makes high resolution mosaics of the rover workspace and surroundings, however, SuperCam is only able to acquire one or two targets per stop (if any at all). As such, a workspace may have 15 ridges present that are seen in the Mastcam-Z images, but if we only have 1 SuperCam target in the plan and the priority is to characterize the bulk bedrock composition, a ridge target may not be selected. We have tried to emphasize this in the text in the Figure caption and Methods section as well.

• Include N arrow on maps, and orbitally defined unit colors need a key of their own.

We have added these into Figure 1 as recommended.

• Line 77- olivine-bearing rocks at Mast Peak (where is this on a map? Maybe I missed it).

We have also added this into Fig.1.

• Line 106 and lines 633-635: The unsupervised model yielded 8 clusters there have been more/less if it was a supervised model vs. the unsupervised?

We think the reviewer is asking if there would be more/less clusters if it was a supervised model. We do not believe there would be much deviation from the number of clusters used given their rather clear visible separation, especially in Figs. 2C and 2D. One thing that could have been changed is grouping the two carbonate clusters and the two high silica clusters, (or removing the high Mg, high Si cluster for the East and West Margin targets that are likely a mix of olivine and silica). We ultimately decided to use an unsupervised method to minimize human bias from the data analysis, especially given the unknowns involved in Mars exploration and the fact that SuperCam is able to target a greater diversity of rock types in the area compared to other instruments on the rover arm. For example, it is uncertain whether a supervised model would have recognized the high Fe targets due to their small number and due to the mission being unable to deploy the proximity science instruments on the Northern Margin unit.

• Line 108- what is a "silhouette score"; please make sure you expand on the terminology, not everyone will know what this is.

We have added a description of the silhouette scores in the Methods section and detailed this in the main text so it is easier for the readers to understand.

• Figure 2 – are the error bars on this data less than the symbols?

Yes they are, and we have added the description in the figure caption as recommended.

• Lines 469-470; where is the fluorite coming from? What is the source of fluoride?

This is something that we currently do not know the answer to. There have been aqueous alteration studies in Gale crater of fluorine enrichment in aqueous systems along Curiosity's traverse. It is possible that we have a similar process occurring in Jezero, but we currently do not have any clear high-F sources. Hopefully, we find some with our continued exploration of the crater rim once we wrap up our investigations at the Lac de Charmes area.

• Line 498 – what is the mechanism that is specifically responsible for the pH to drop?

With lacustrine systems, there are many factors that could cause a change in pH such as changes in groundwater or meteoric water input, changes in climate, or changes in evaporation rate (e.g., transition to an arid environment). From our analysis, we cannot pinpoint which could be the cause, but mention these possibilities in the Fig. 10 caption.

Response to reviewers:

Reviewer #1 (Remarks to the Author):

Review of "Investigating the origin and lake-associated alteration of the olivine-rich Margin unit in Jezero crater, Mars" by Bedford et al.
Brian Hynek
University of Colorado

My comments have been sufficiently addressed, and I think the authors did a fine job addressing the other referee's suggestions as well. I am mostly pleased with the state of the manuscript. There are some editing issues to address. Repeatedly in the text and also figure captions, the authors talk about the carbonate-rich fluids forming ridges (e.g., line 436, Figure caption 10, and beyond). The minerals were precipitated in fractures in the bedrock. Presumably, later differential erosion led to the harder materials to become manifest as ridges. This need to be clarified. Supplementary materials have an incomplete author list: "...Adrian Brown, Erwin Dehouck, anyone else? Katie Stack, Susanne Schroder?" There are other minor editorial issues throughout, so a thorough review by the authors is warranted.

We thank the reviewer for their helpful comments throughout this process. We have looked through the manuscript one more time and clarified that the ridges formed from fracture fill that later experienced differential erosion. We also checked for editing issues, and made sure the supplementary materials has the same manuscript title and author list as the main manuscript.

Reviewer #2 (Remarks to the Author):

The manuscript edits are reasonable support the proposed major claims, and therefore I recommend publication of this manuscript.

Thank you

We are grateful for this reviewers comments and glad that they are satisfied with the changes.
