# Supplementary material for: Lake- and groundwater-associated alteration of the olivine-rich Margin unit in Jezero crater, Mars
Source: Commun Earth Environ. 2026 Sep 21;7(1):728. doi: 10.1038/s43247-026-03997-9 (PMC13593571; doi:10.1038/s43247-026-03997-9)
Supplement: Supplementary file 2 [file 43247_2026_3997_MOESM2_ESM.pdf]

Supplementary information:
Lake- and groundwater-associated alteration of the olivine-rich Margin unit in Jezero crater, Mars.

Candice C. Bedford, Eleni Ravanis, Roger C. Wiens, Elise Clavé, Julene Aramendia, Juan Manuel Madariaga, Eleanor Moreland, Stephanie Connell, Alexander Jones, Briony Horgan, Olivier Forni Bradley Garczynski, Susanne Schröder, Kenneth Williford, Linda Kah, Kathryn Stack, Lucia Mandon, Agnés Cousin, Pierre Beck, Erwin Dehouck, Clément Royer, Adrian Brown

Supplementary Materials 1: Cluster results

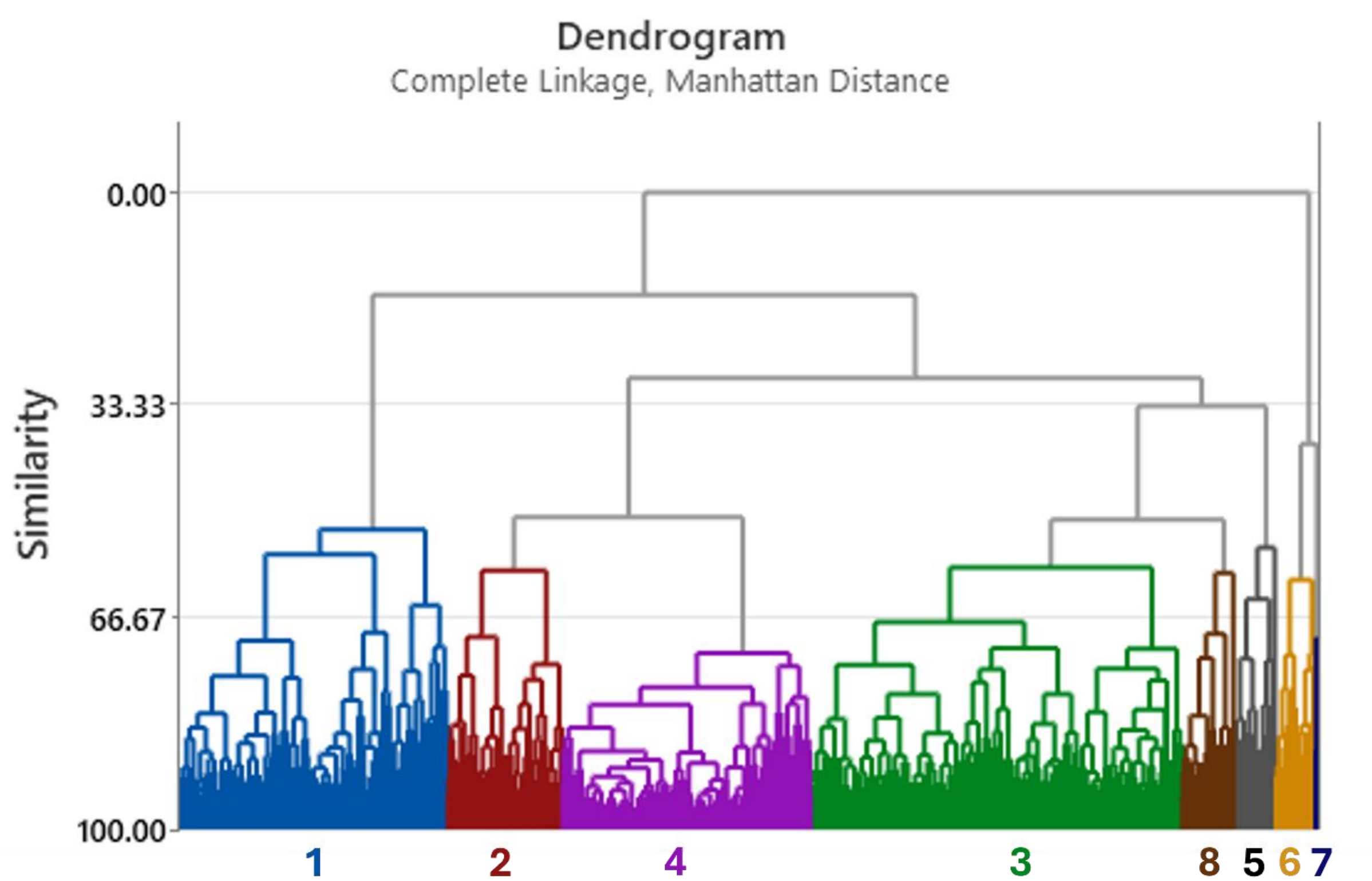

Figure S1: Dendrogram of cluster results with number of observations on the x-axis and Similarity level on the y-axis.

Table S1: Measured mean long axis grain sizes for SuperCam targets in the Margin Unit across different textures compared to those measured in previous units of the Western fan and Séitah on the crater floor.

Target	Class/Unit	Count	Mean	StDev	Elevation	Reference
Bullvalley	Matrix-supported	141	0.44	0.19	-2401	this paper
Gray_Peak	Matrix-supported	200	0.57	0.26	-2371	this paper
Lake_Wyodaho	Matrix-supported	200	0.39	0.22	-2372	this paper
Naronga	Matrix-supported	200	0.68	0.32	-2406	this paper
Nymph_Lake	Matrix-supported	200	0.46	0.30	-2383	this paper
Baresand_Point	East Granular	240	0.70	0.33	-2408	this paper
Peak_Island	East Granular	200	0.54	0.21	-2423	this paper
Purdy_Point	East Granular	203	0.42	0.22	-2423	this paper
Red_Bluff	East Granular	200	0.43	0.24	-2423	this paper
Sinking_Ship	North Granular	200	0.68	0.31	-2350	this paper
Tanner_Graben	High Granular	200	0.73	0.36	-2204	this paper
White_Butte	High Granular	90	1.22	0.59	-2298	this paper
Woolsey_Butte	High Granular	208	0.88	0.57	-2337	this paper
Point_Maud	Rubbly	300	0.87	0.94	-2423	this paper
Frying_Pan_Spring	Rubbly	200	1.02	1.06	-2380	this paper
Solitude Lake	Western Fan	442	0.25	0.12	-2449	Ives et al. (2025)
Solva	Western Fan	445	0.23	0.17	-2424	Ives et al. (2025)
Berry Hollow	Western Fan	156	0.08	0.028	-2523	Stack et al. (2024)
Pignut Mountain	Western Fan	147	0.07	0.021	-2523	Stack et al. (2024)
Uganik Island	Western Fan	140	0.10	0.092	-2517	Stack et al. (2024)
Thornton Gap	Western Fan	129	0.51	0.497	-2520	Stack et al. (2024)
Novarupta	Western Fan	154	0.08	0.024	-2526	Stack et al. (2024)
Moody Creek	Western Fan	40	0.64	0.337	-2535	Stack et al. (2024)
Cape Gull	Western Fan	45	0.59	0.245	-2526	Stack et al. (2024)
Issole	Séitah	141	1.13	0.19	-2574	Wiens et al. (2022)
Estoublaisse	Séitah	140	1.57	0.15	-2574	Wiens et al. (2022)
Bezaudun	Séitah	180	1.35	0.18	-2574	Wiens et al. (2022)
Sagnes	Séitah	115	1	0.2	-2574	Wiens et al. (2022)
Monier	Séitah	97	1.49	0.26	-2573	Wiens et al. (2022)
Mairola	Séitah	108	1.52	0.29	-2573	Wiens et al. (2022)
Cine	Séitah	95	1.57	0.28	-2573	Wiens et al. (2022)
Norante	Séitah	143	1.62	0.17	-2574	Wiens et al. (2022)
Brac	Séitah	117	1.62	0.2	-2569	Wiens et al. (2022)
Pierlas	Séitah	137	1.5	0.29	-2569	Wiens et al. (2022)
Ferres	Séitah	112	1.45	0.16	-2569	Wiens et al. (2022)
Ubraye	Séitah	126	1.58	0.18	-2568	Wiens et al. (2022)

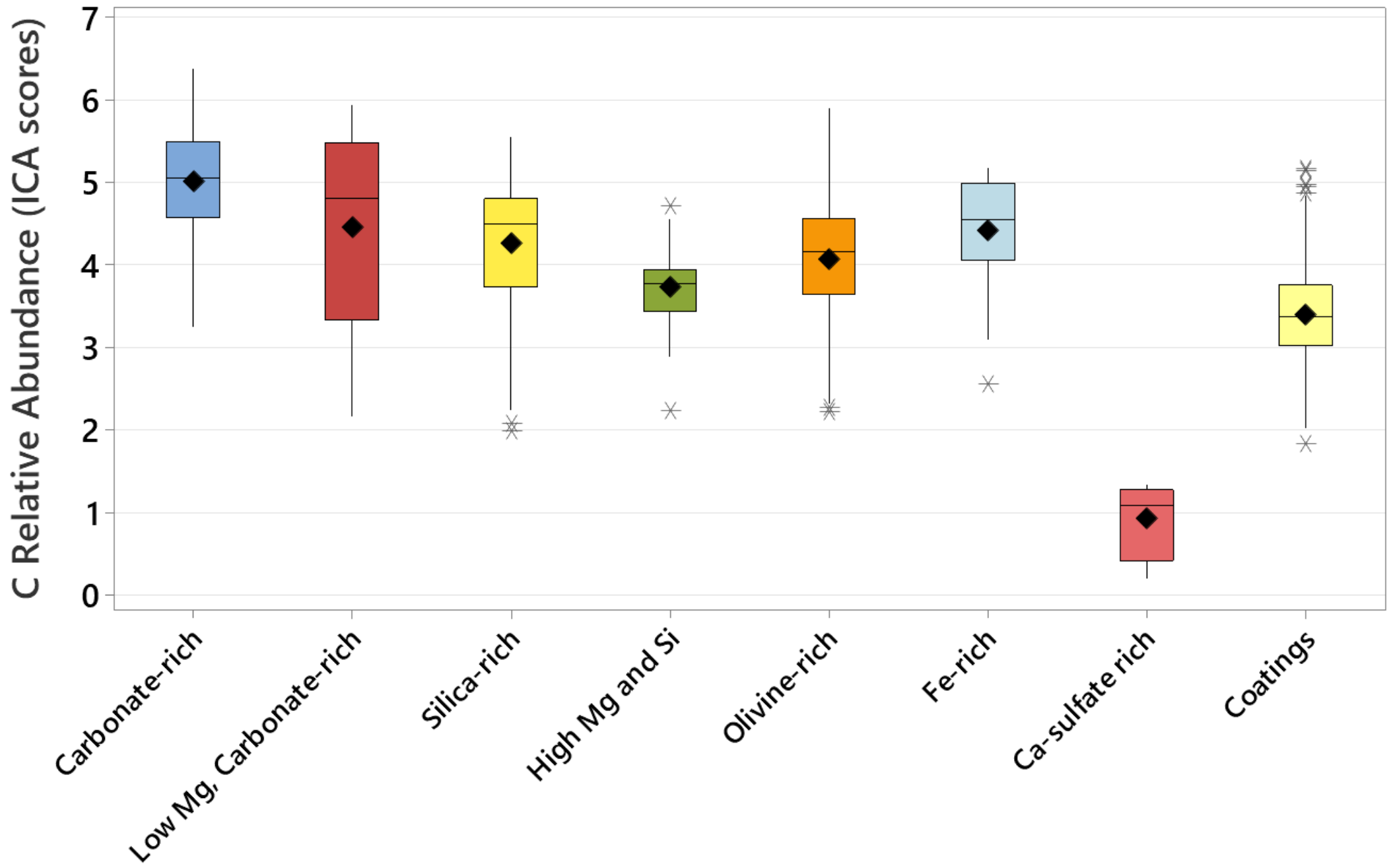

Figure S2: Box and whisker plots of C LIBS ICA (Independent Components Analysis) scores with black diamonds showing mean scores and asterisks representing outlying scores. Carbon ICA scores can be negatively correlated with distance to the target. As such, we provide boxplots for the target distances for each cluster below (Fig. S3) to highlight that the higher C scores of the Carbonate-rich cluster are not likely a result of a smaller distance to the target compared to other clusters.

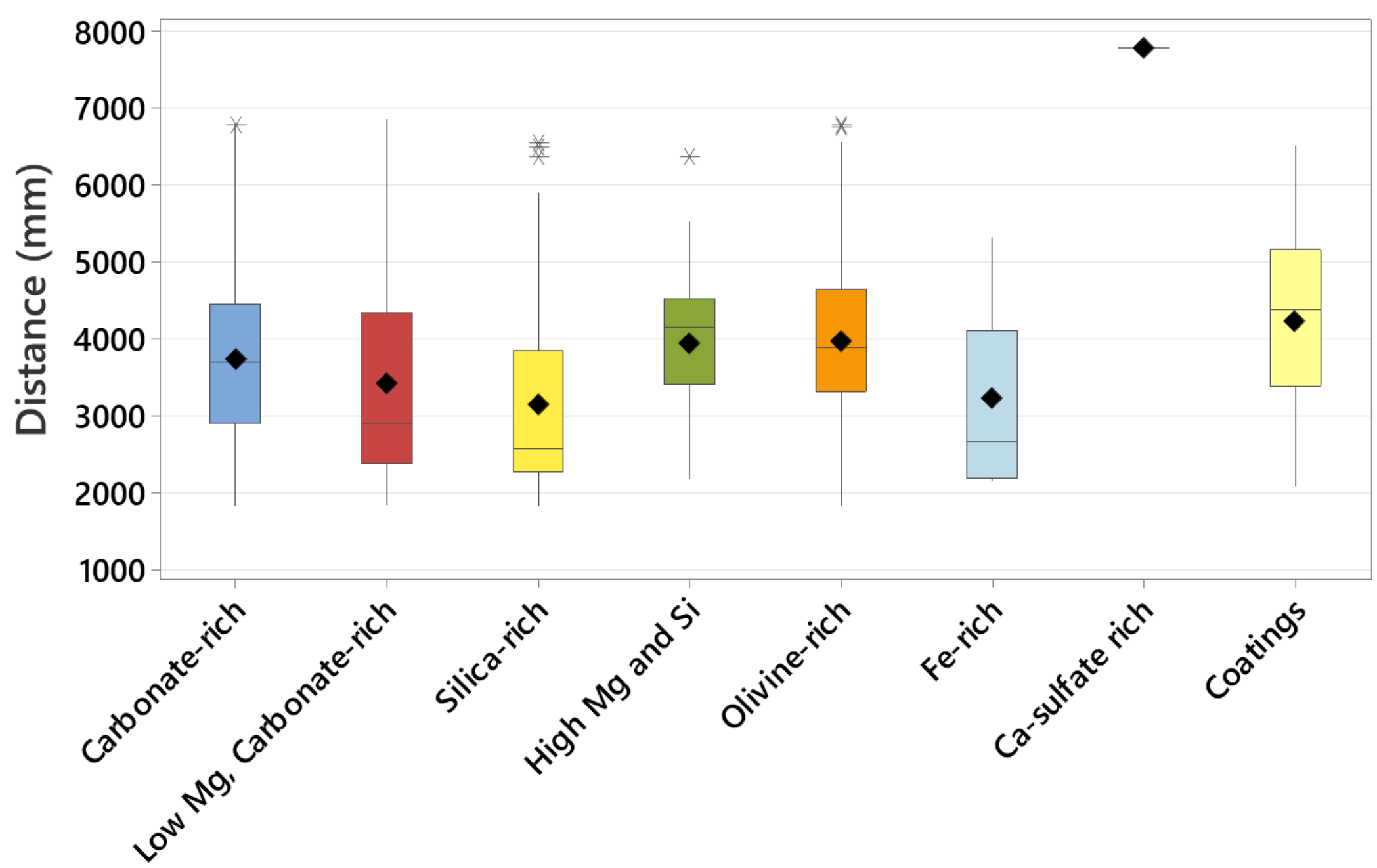

Figure S3: Box and whisker plots of target distances for each cluster as a comparison to ICA scores which may be impacted by this variable (e.g., C).

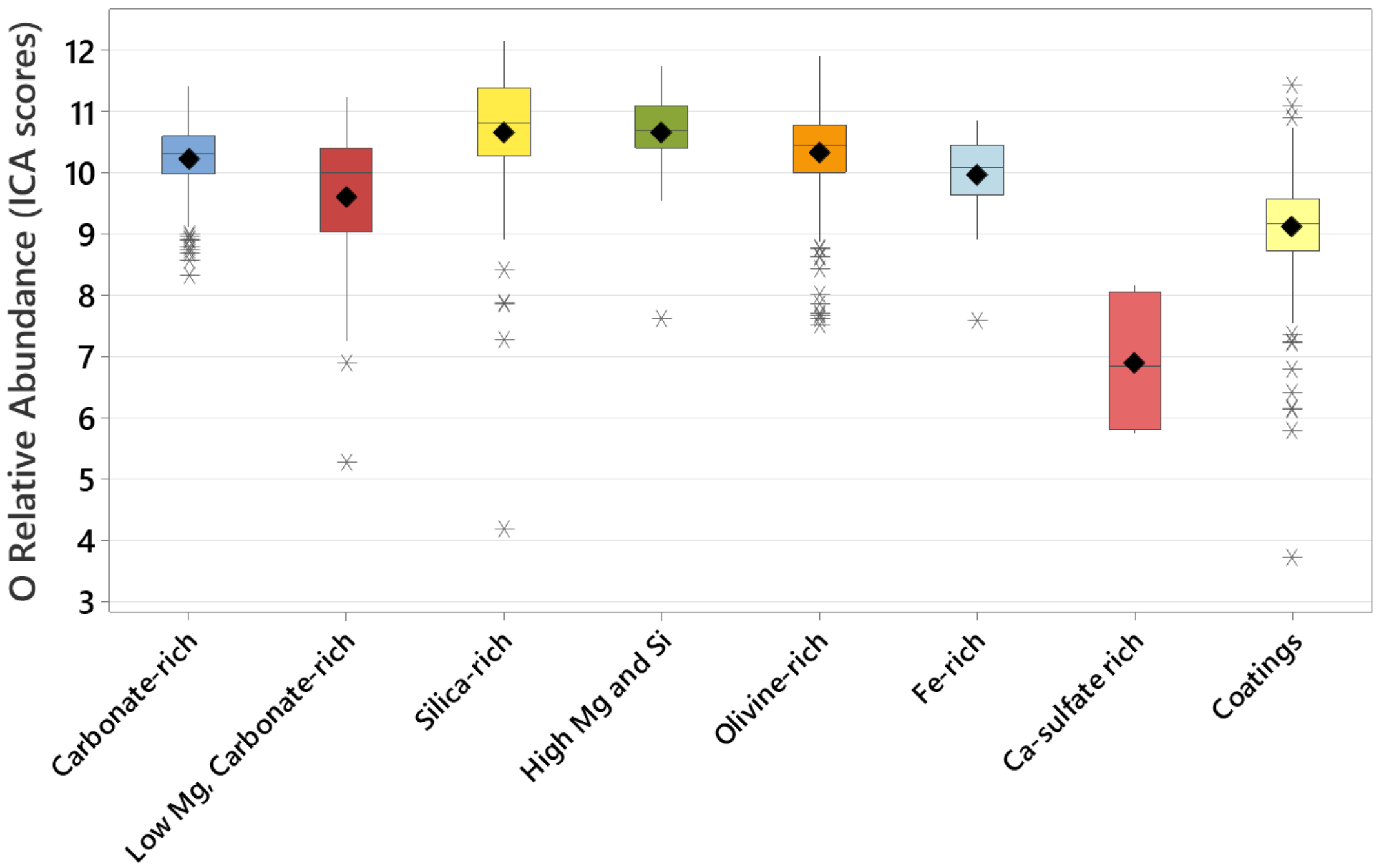

Figure S4: Box and whisker plots of O ICA scores with black diamonds showing mean scores and asterisks representing outlying scores.

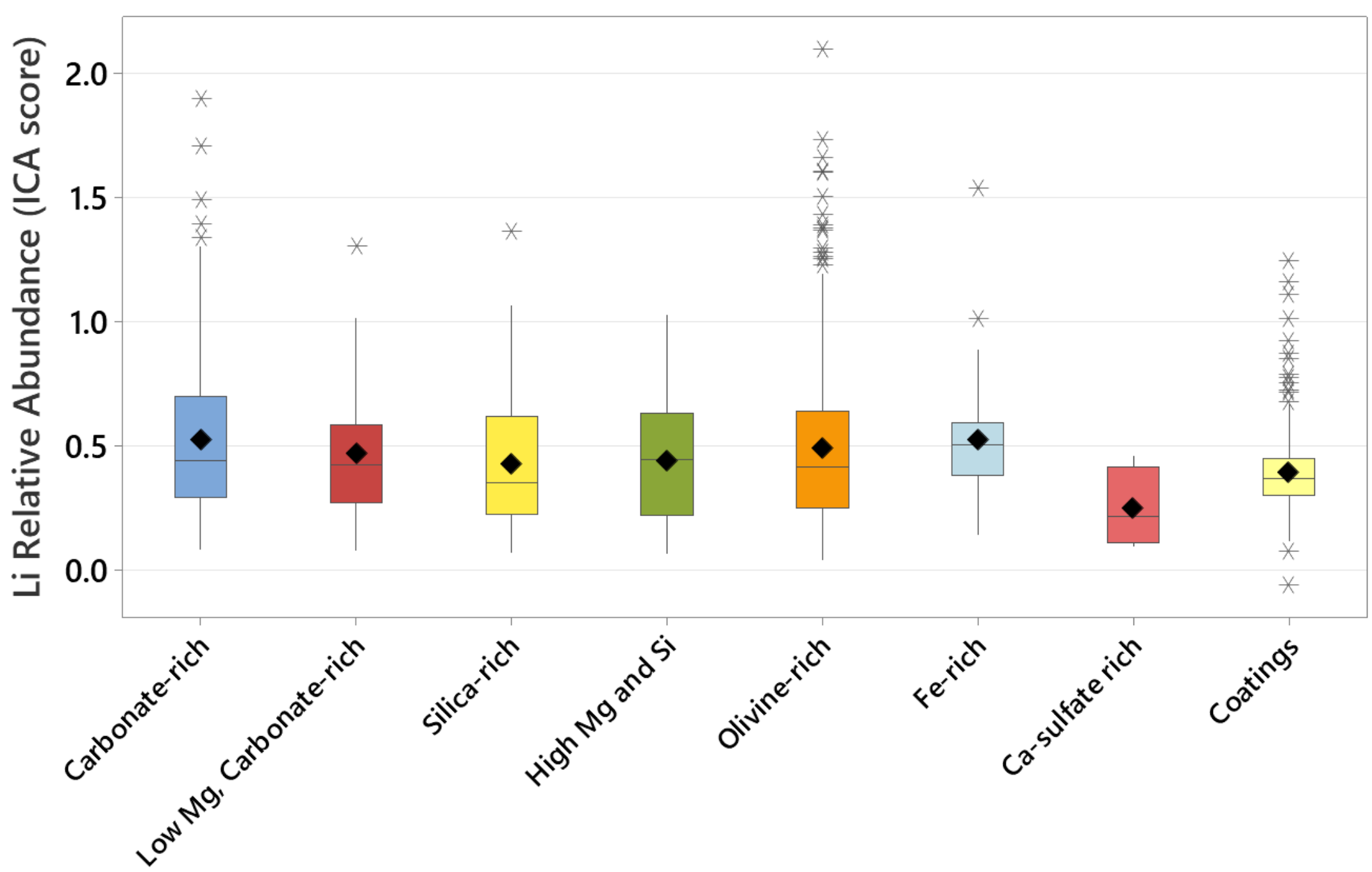

Figure S5: Box and whisker plots of Li ICA scores with black diamonds showing mean scores and asterisks representing outlying scores.

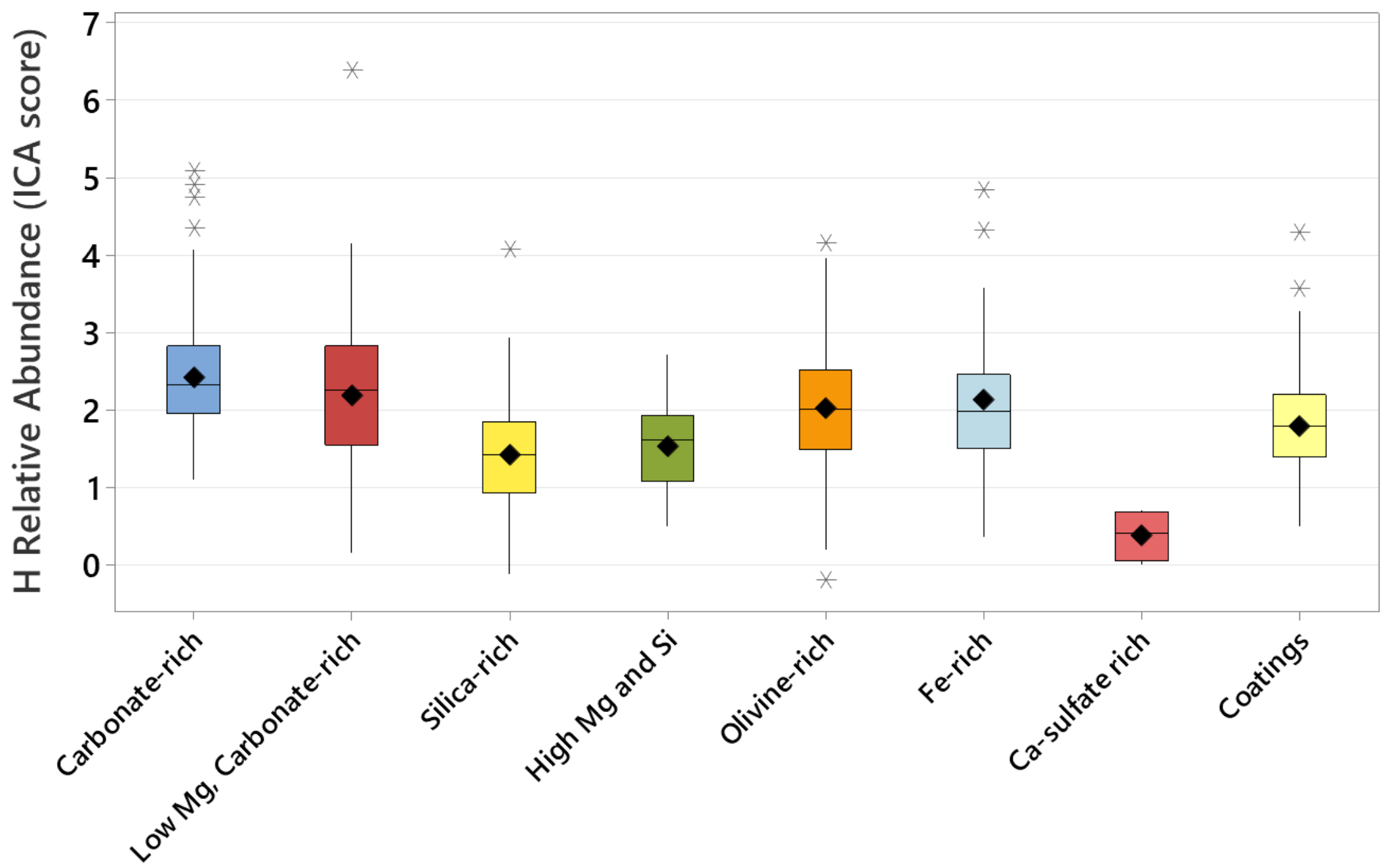

Figure S6: Box and whisker plots of H ICA scores with black diamonds showing mean scores and asterisks representing outlying scores.

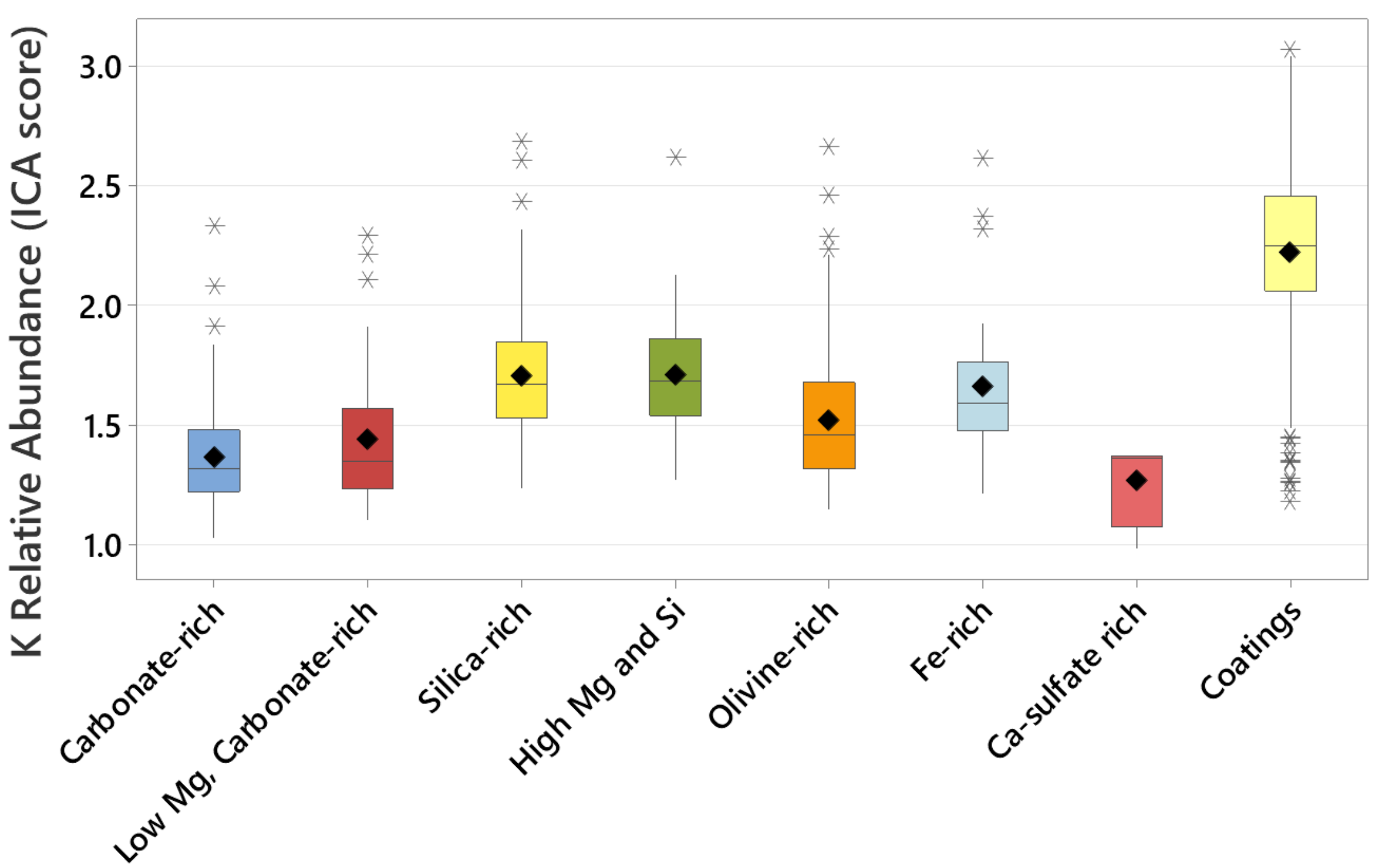

Figure S7: Box and whisker plots of K ICA scores with black diamonds showing mean scores and asterisks representing outlying scores.

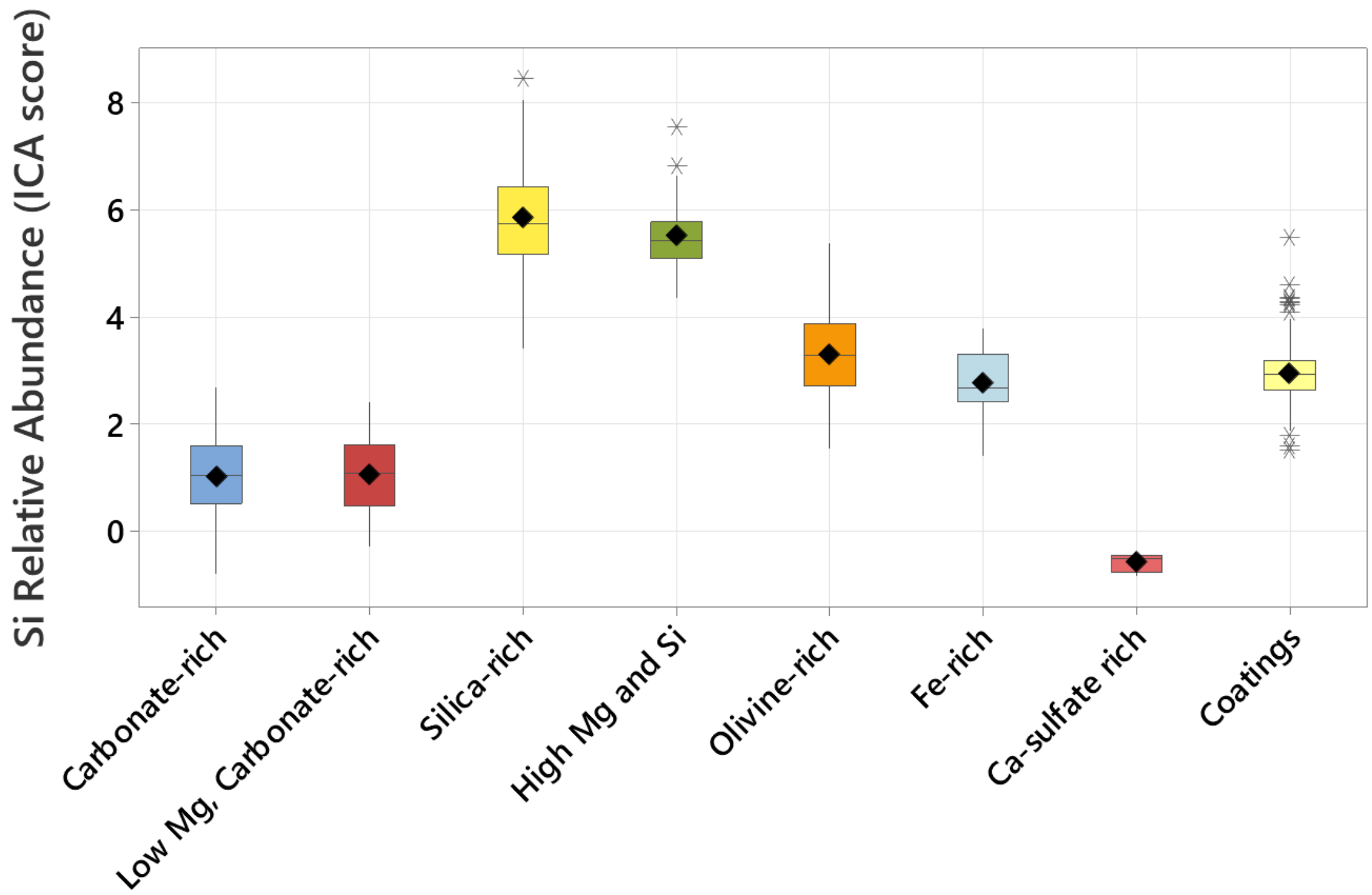

Figure S8: Box and whisker plots of Si ICA scores with black diamonds showing mean scores and asterisks representing outlying scores.

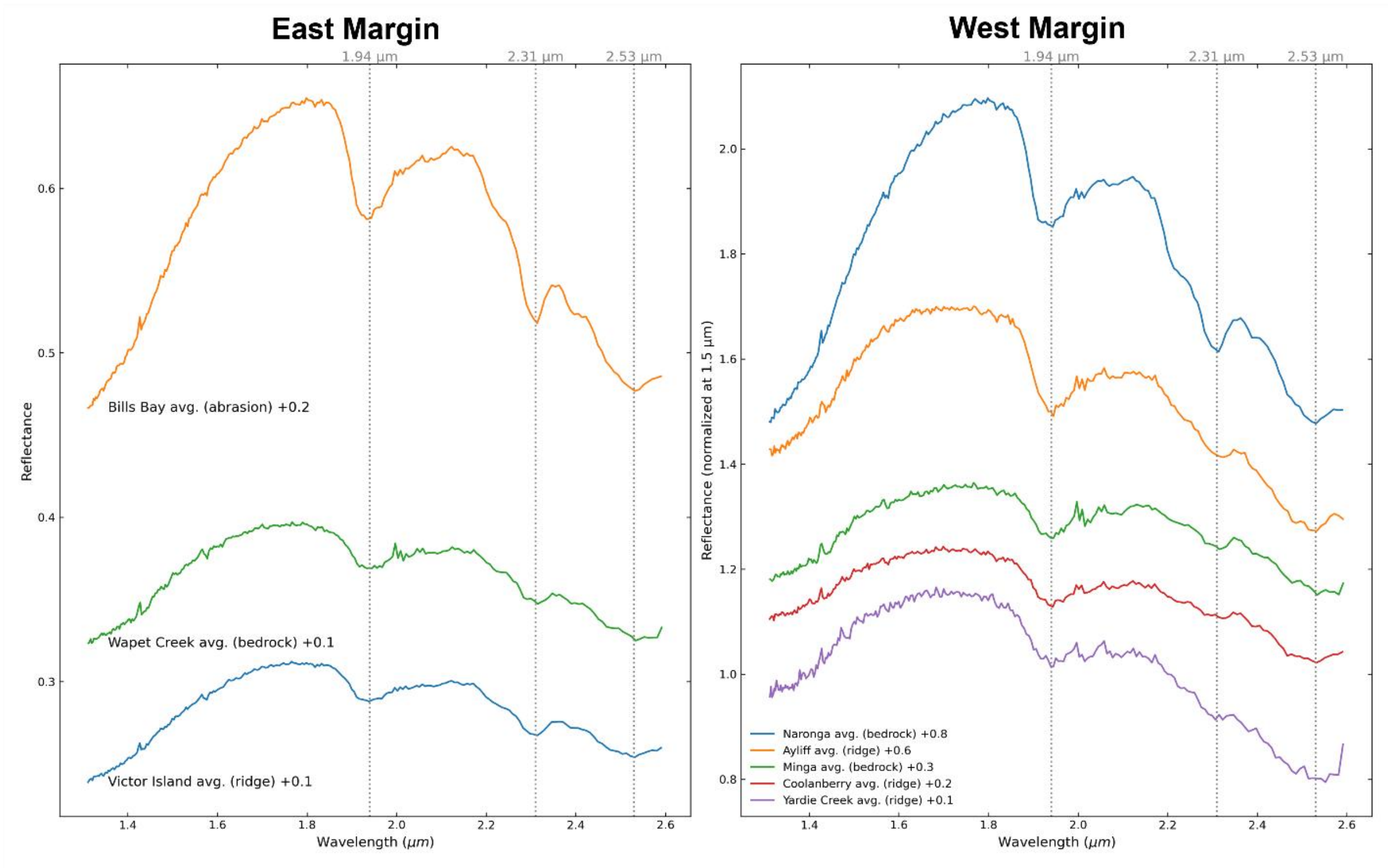

Figure S9: SuperCam infrared reflectance spectra (1.3 – 2.6 µm) shows the comparison of ridge features to the surrounding bedrock in the East and West Margin. The East Margin is comprised of various targets such as the Bills Bay abrasion patch, Wapet Creek natural surface bedrock (non-abraded), and Victor Island classified as a type 1 ridge. The West Margin comprises the Naronga and Minga natural surface bedrock targets, and Yardie_Creek (type 1), Coolanberry (type 2), and Ayliff (type 2) ridges. Infrared spectra for some targets show a steep slope at shorter wavelengths from 1.3 – 1.8 µm indicative olivine or Fe-bearing carbonate. Some spectra exhibit variable band strength at the 1.94 µm H_2O absorption band, and weaker bands at 2.31 µm and 2.53 µm bands indicative of Fe/Mg-phyllosilicates and/or carbonates. The 2.53 µm carbonate bands are weaker in some targets, which may be a factor of variable dust cover, coating and different lighting conditions.

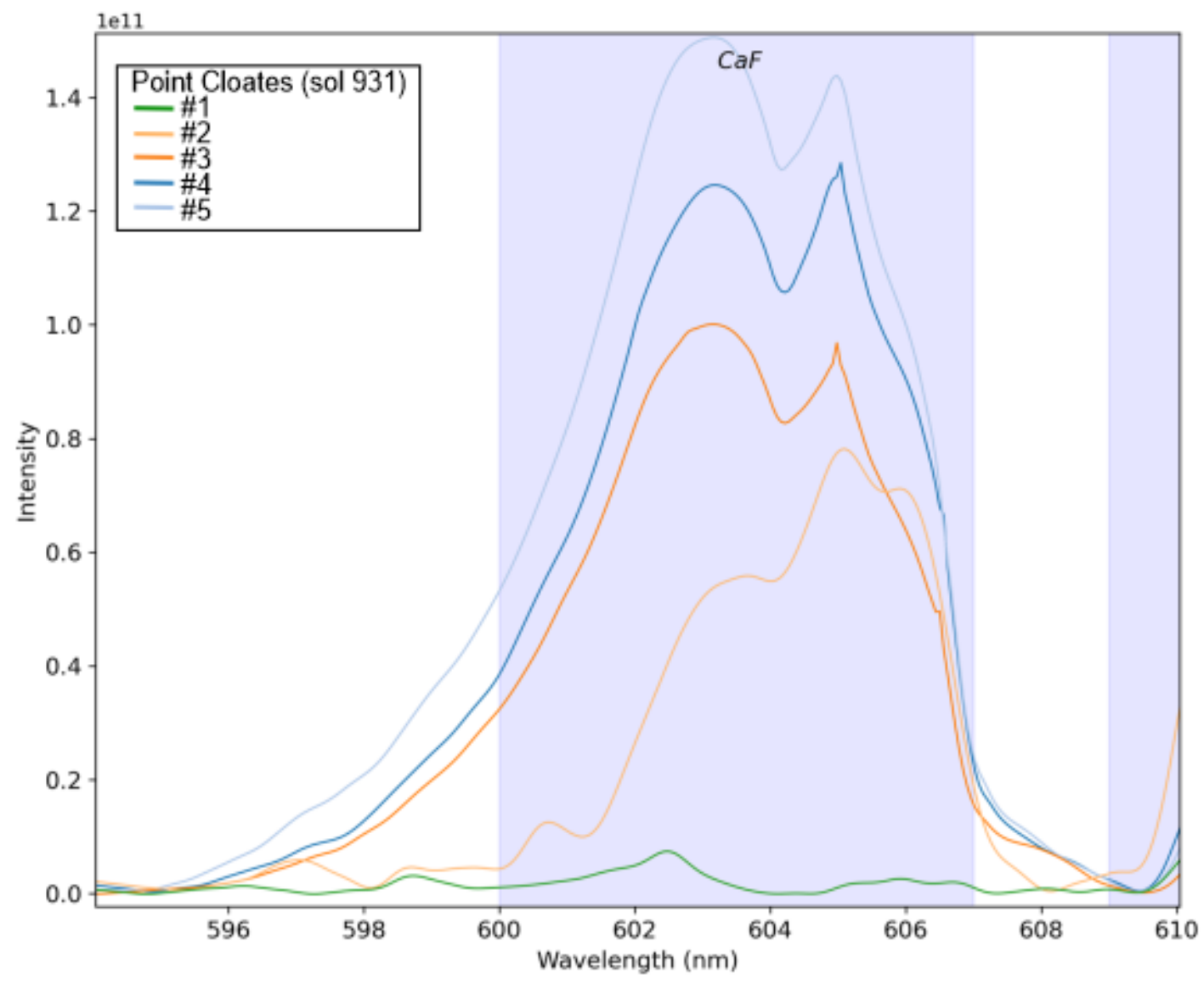

Figure S10: Ca-F molecular bands in the SuperCam Point Cloates target LIBS data for each point in the target raster.

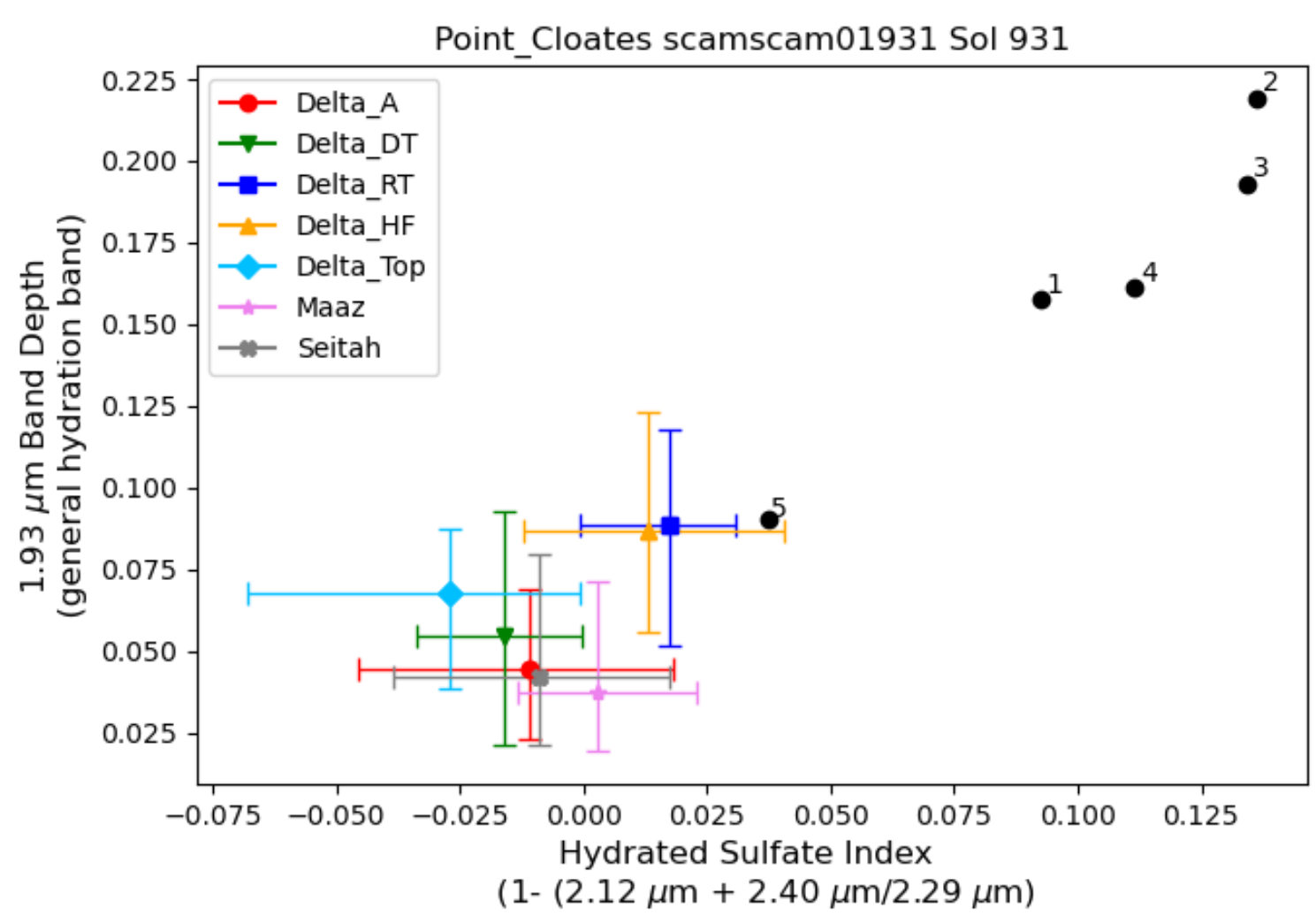

Figure S11: IR Spectral parameter plot for the Point Cloates target showing the hydrated sulfate index (1- (2.12 μm + 2.40 μm/2.29 μm) against the 1.93 μm band depth parameter. Reference points are provided for units on the crater floor (Séítah and Máaz) and those of the Western Fan (Delta). Delta_A includes targets from the Amalik unit, Delta_DT includes targets from the Devils Tanyard unit, Delta_RT from Rocky Top, HF from Hogwallow Flats, and Delta_Top are targets acquired on the Upper Fan in the Tenby formation. Numbered points represent each raster point in the Point Cloates target.

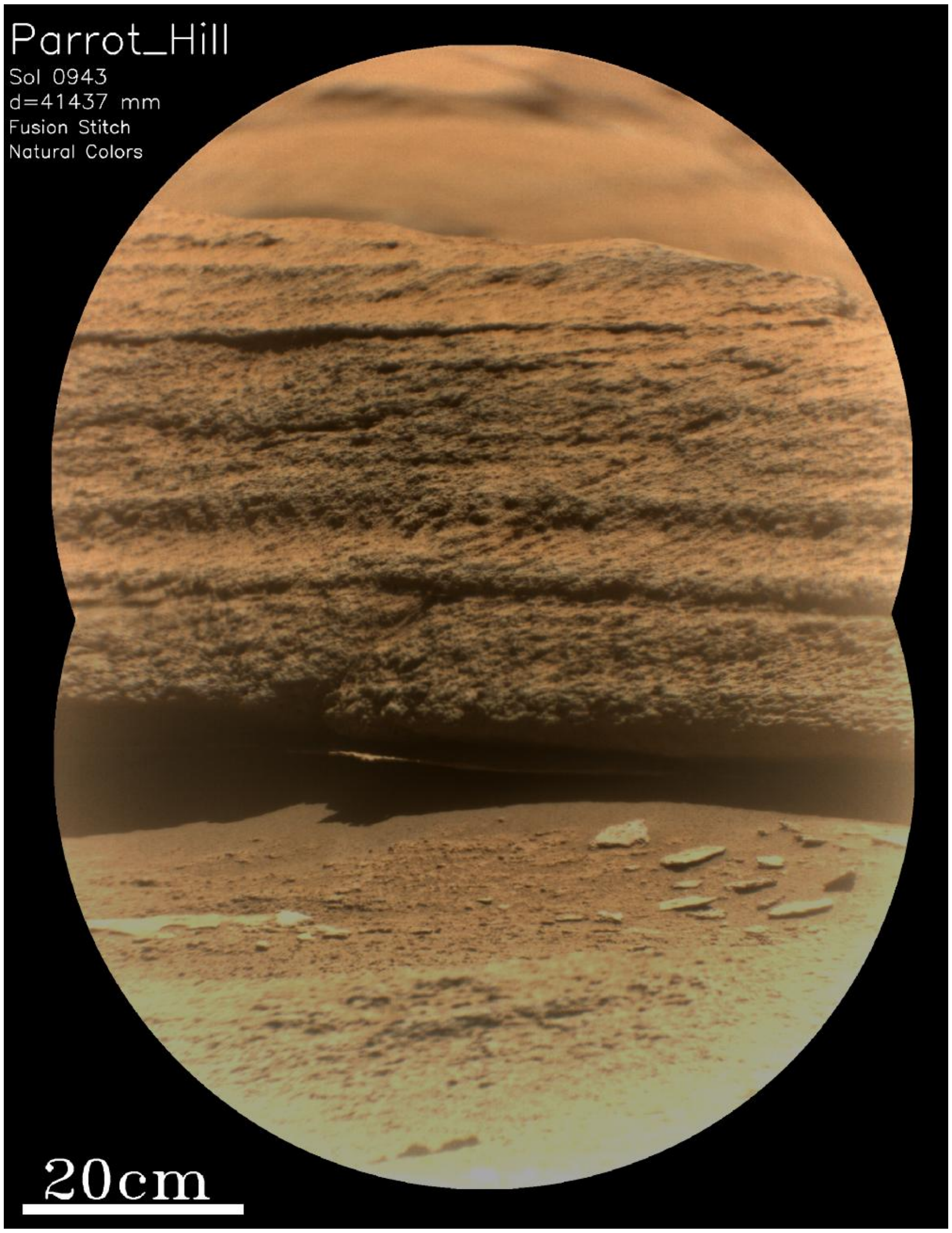

Figure S12: Parrot_Hill long distance RMI observation of the East Margin Unit showing possible grading.

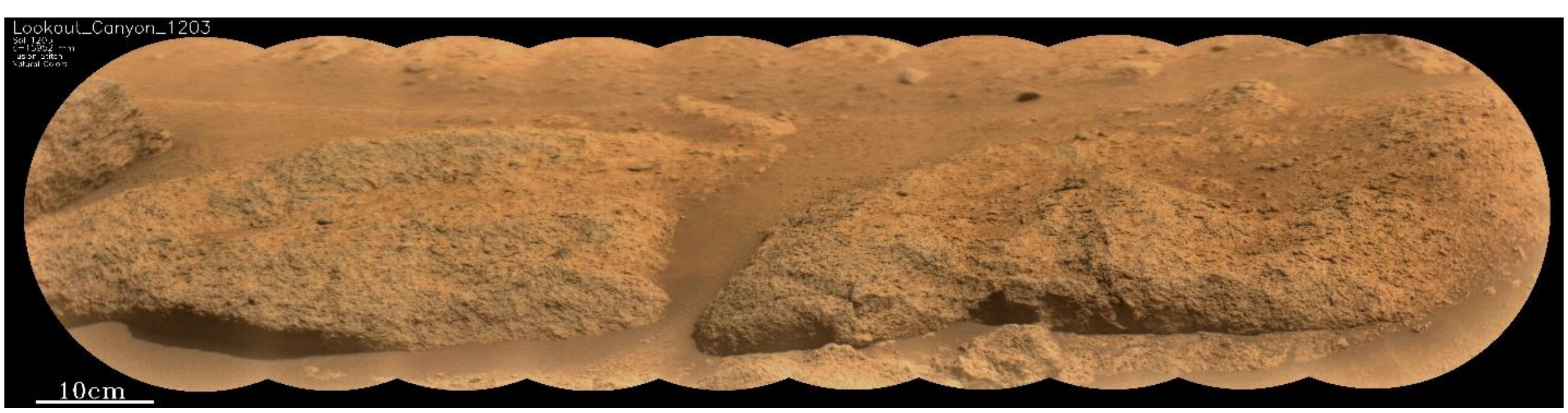

Figure S13: Lookout_Canyon long distance RMI observation of a layer in the North Margin Unit.

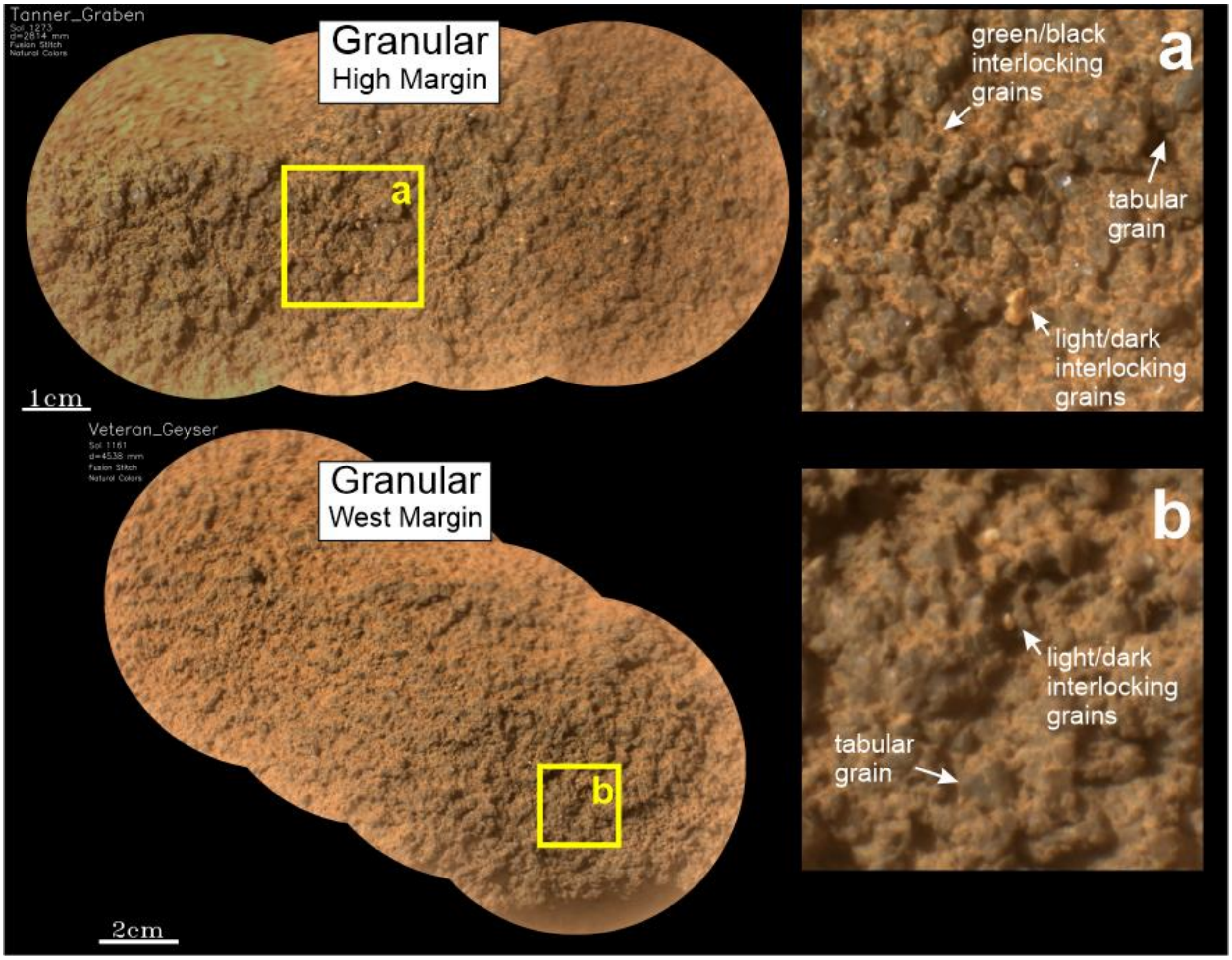

Figure S14: Comparative figure showing similarities between the Granular West Margin target (Tanner Graben) and High Margin target (Veteran_Geyser) RMIs. Close-up images (a and b) are both 2 cm across.

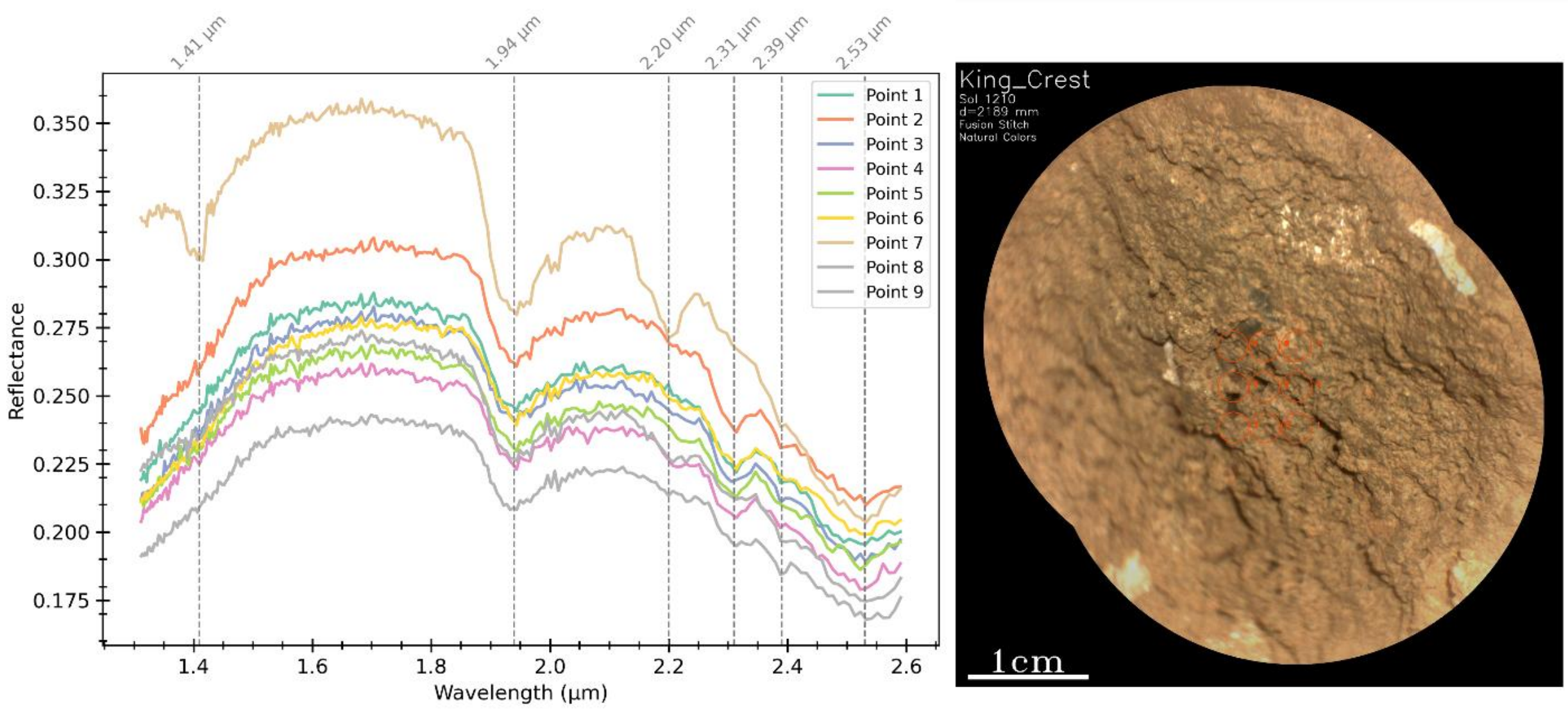

Figure S15: King Crest SuperCam target IR data and IR footprints on the associated target image. Point 7 shows a doublet at 1.4 µm (H_2O and metal-OH), in addition to deep bands at 1.9 µm (H_2O) and 2.2 µm (Si-OH and Al-OH) indicative of kaolin (Al-phyllosilicate). In the RMI image, the IR footprint of point #7 clearly includes a light-toned area of the rock at the edge. The LIBS point of this target did not see chemical data indicative of kaolin here, however, the LIBS footprint is much smaller than that of the IR and it likely missed the light-toned area.

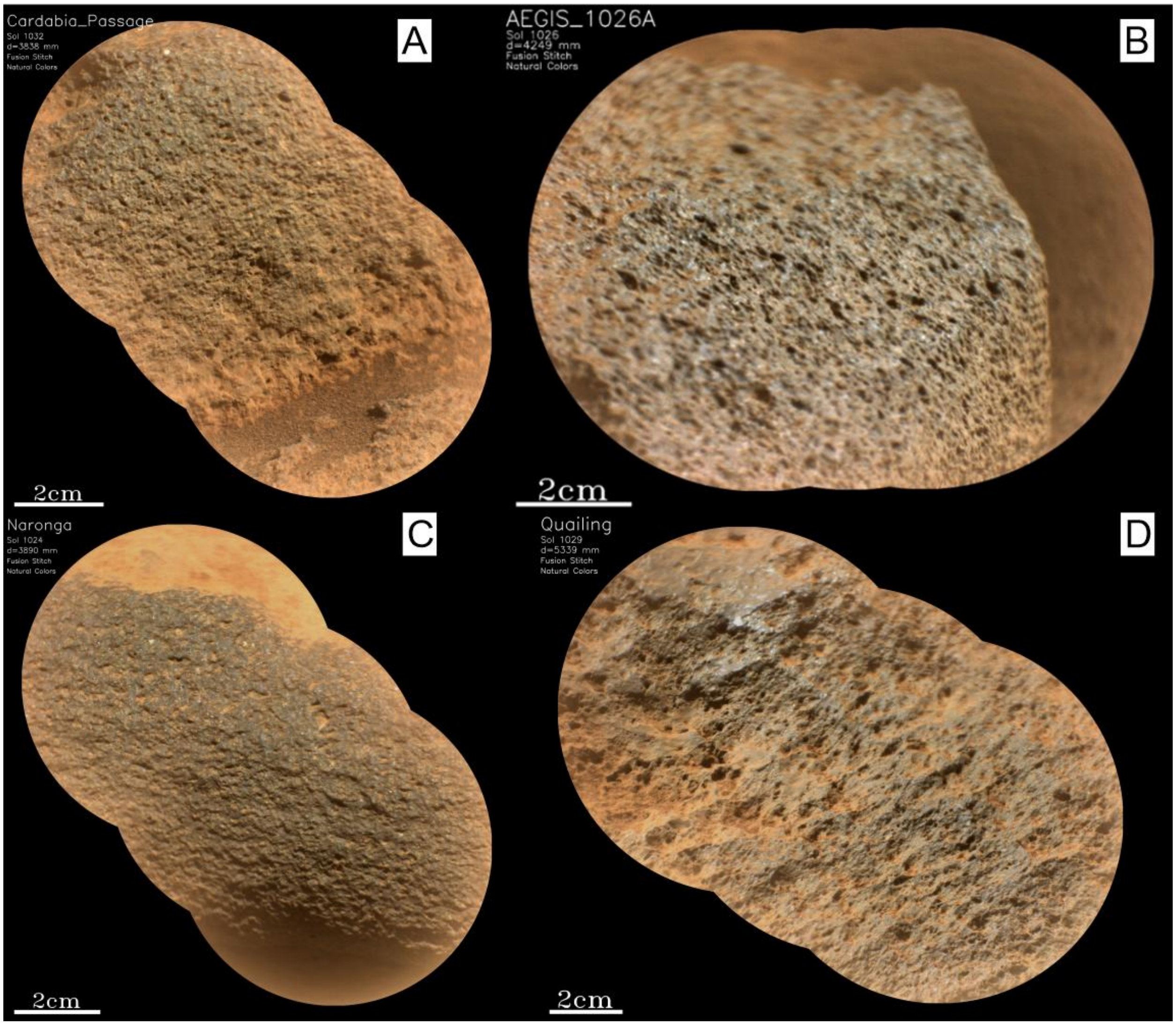

Figure S16: Examples of SuperCam Margin Unit targets that display pits that may be indicative of secondary porosity or physical weathering by the wind. All examples are found in the West Margin. Targets are; A) Cardabia Passage, B) AEGIS 1026A, C) Naronga, D) Quailing.

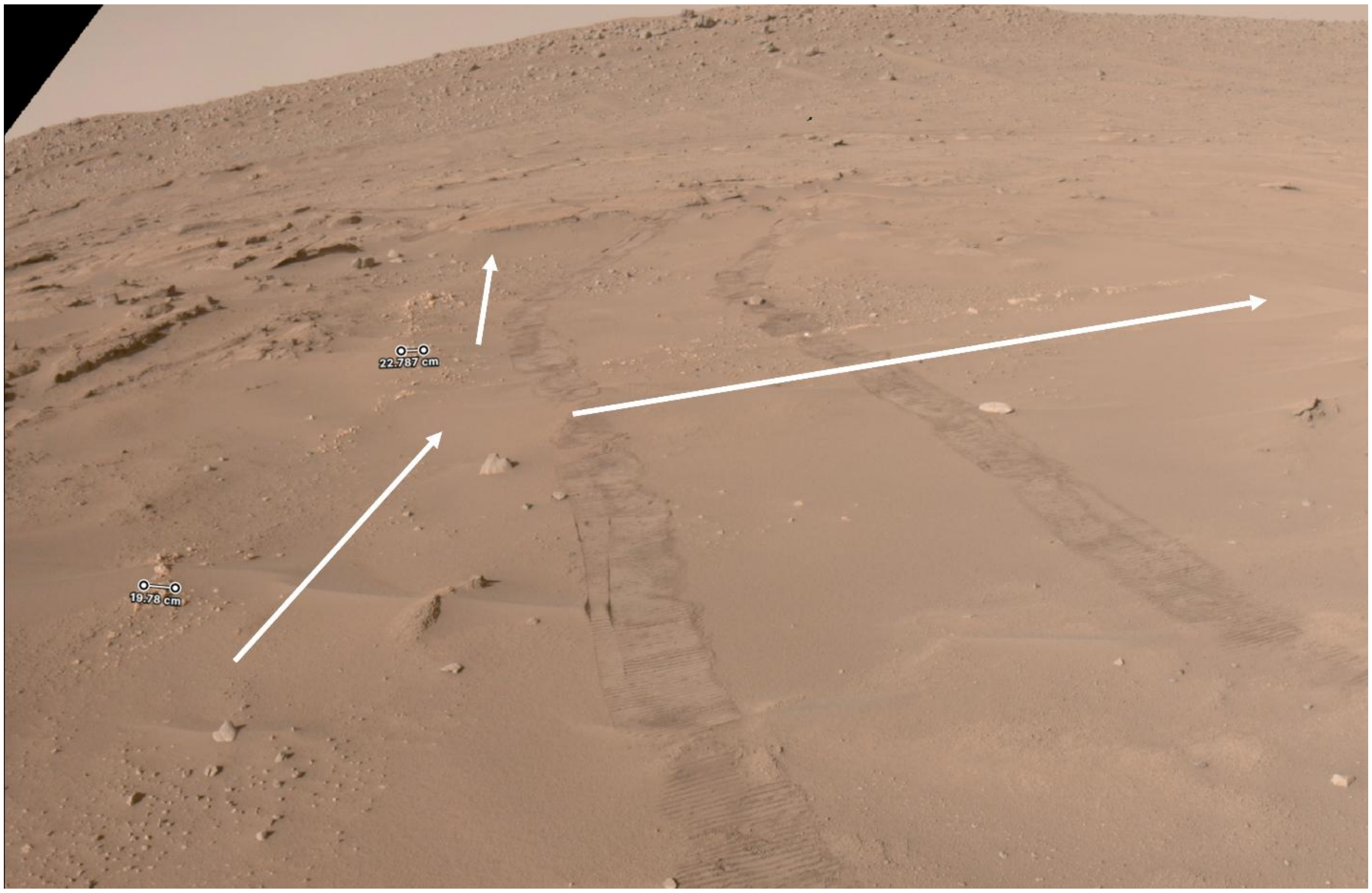

Figure S17: Left Navcam image of the Point Cloates feature extending to the NE and E, highlighted by white arrows parallel to eroded vein feature (Navcam image ID: NLF_0930_0749511621_425RAS_N0452198NCAM03930_0A1195J02, Sol 930).
